# Cadmium-doped molybdenum nanospheres as emerging energy and environmental electrode materials

**DOI:** 10.1039/d6na00770h

**Published:** 2026-10-01

**Authors:** Muhammad Arshad Kamran, Areej Rani, Wasif Ali, Mansour Alhabradi, Li Chen, Hui Qi Wong, Hung-Ju Yen

**Affiliations:** a Department of Physics, University of Okara Okara Pakistan m.kamran@uo.edu.pk; b Institute of Chemistry, Academia Sinica 128 Academia Road, Section 2 Nankang Taipei 11529 Taiwan; c Department of Physics, College of Science, Majmaah University Majmaah 11952 Saudi Arabia M.alhabradi@mu.edu.sa

## Abstract

Cadmium-doped strontium molybdate (CSMO) nanospheres were synthesized and systematically evaluated for bifunctional applications in energy storage and photocatalysis. The optimized CSMO-9% exhibited uniform morphology, reduced band gap, and enhanced conductivity. Superior electrochemical performance and remarkable photocatalytic dye degradation highlight CSMO's potential as an efficient, multifunctional material for sustainable energy and environmental applications.

## Abstract 2

Cadmium-doped strontium molybdate (CSMO) nanospheres were synthesized and systematically evaluated for bifunctional applications in energy storage and photocatalysis.

## Graphical abstract

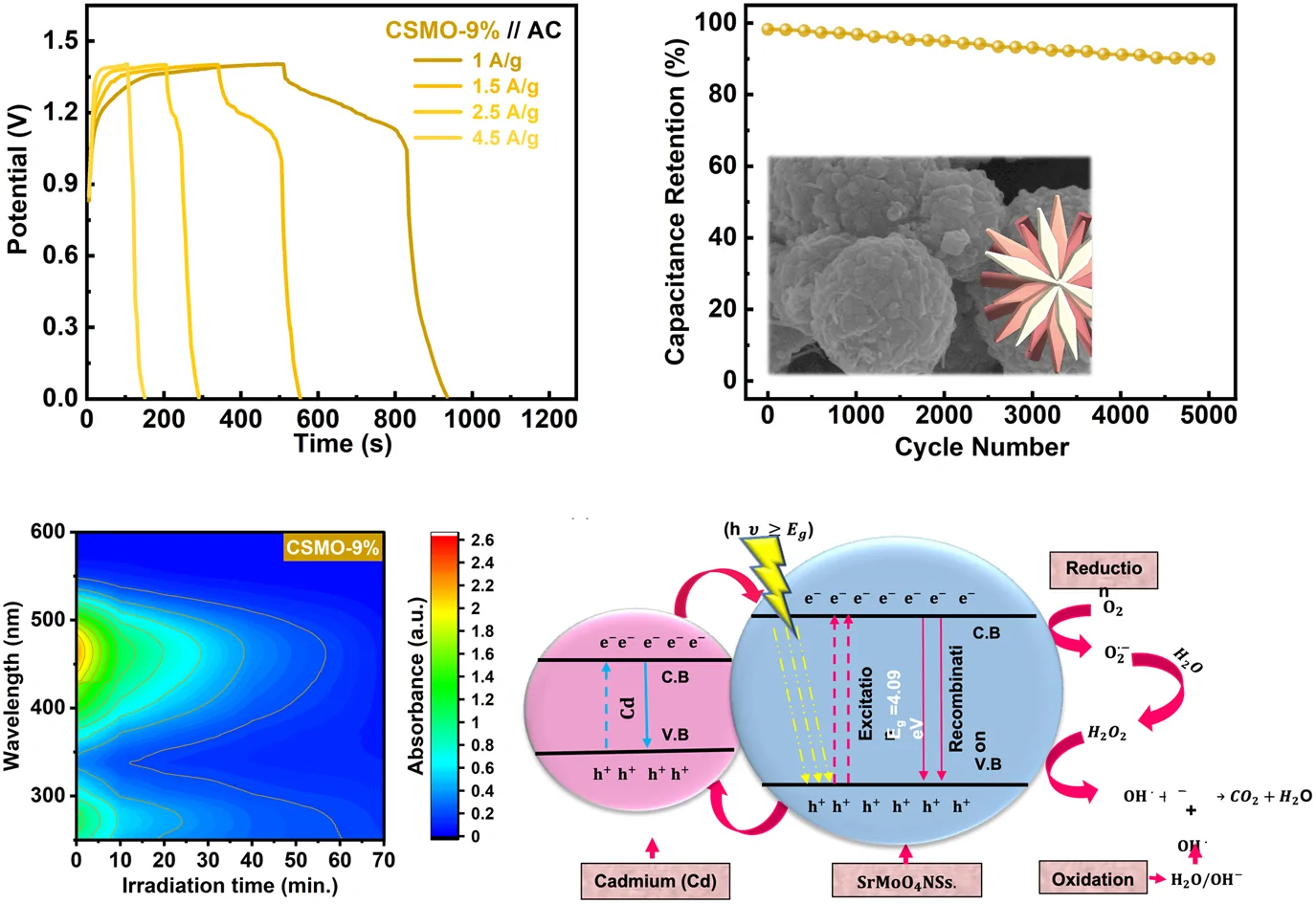

## Introduction

Since the beginning of the 21st century, contemporary technologies based on fossil fuels have created a variety of major environmental issues. Green energy generation, such as solar and tide energy, has emerged as an important study area for long-term and sustainable energy development in contrast, these renewable energy sources are distinguished by their location, performance, and stability.^1^ Electrochemical energy storage systems constitute a foundational component of sustainable energy technologies, enabling the effective management and utilization of renewable power sources. Their deployment alleviates dependence on fossil-based energy and contributes to mitigating environmental burdens. Among various storage modalities, supercapacitors (SCs) have emerged as a focal point of research interest alongside batteries and fuel cells. SCs are distinguished by their high power output, rapid charge–discharge kinetics, long cycling stability, and minimal maintenance requirements. Their low equivalent series resistance and lightweight architecture render them suitable for portable and high-power applications, thereby serving as an intermediate technology bridging the performance gap between conventional capacitors and rechargeable batteries.^2–4^

SCs are categorized into electric double-layer capacitors (EDLCs) and pseudocapacitors. EDLCs store energy through electrostatic charge accumulation at the electrode–electrolyte interface using high-surface-area carbons such as activated carbon and graphene. Pseudocapacitors, in contrast, rely on rapid, reversible faradaic redox reactions involving materials like metal oxides, hydroxides, or conductive polymers. Although SCs are environmentally friendly and deliver high power, their energy density remains limited by the intrinsic properties of electrode materials. Consequently, research focuses on designing novel electrode architectures and compositions to enhance charge storage and electrochemical performance. Recent advances have concentrated on the creation of nanostructured electrode materials particularly transition metal oxides, carbon-based materials. Furthermore, recent studies have demonstrated that rational design strategies such as heteroatom doping and defect engineering, significantly improve electrochemical activity due to increased surface area, shorter ion transport paths, and enhanced redox reactions. In this regard, pseudocapacitive and hybrid materials have received a lot of attention because they combine quick surface charge storage with a higher energy density than typical capacitive systems.^1^

Transition metal oxide (TMO)-based electrodes have gained particular attention due to their high theoretical capacitance, abundance, and versatile properties. However, their practical use is often restricted by low energy density and poor cycling stability. Thus, ongoing efforts aim to develop alternative, cost-effective materials that balance energy and power densities with improved structural and electrochemical durability.^5–7^

Among TMOs, molybdenum-based compounds have been widely explored as electrode materials for supercapacitors due to their distinctive physicochemical characteristics. The multiple valence states of molybdenum enable rich redox reactions, while their robust structural framework promotes rapid ion diffusion and charge transfer. Furthermore, molybdenum-containing oxides, like NiMoO_4_, CoMoO_4_, MnMoO_4_, FeMoO_4_, CeMoO_4_, ZnMoO_4_, and CuMoO_4_, can serve as a mechanical buffer, mitigating strain and stress accumulation during repeated charge–discharge cycling and thereby enhancing structural integrity and long-term performance.^8–16^ In this regard, strontium molybdate (SrMoO_4_, SMO) is noticeable as a highly capable material as it has been employed in various applications such as optics, fiber lasers, luminescent resources, photocatalysis, scintillation detection, solar cells, fuel cells, electrochemical sensors, electrocatalyst, and SCs due to its notable intrinsic activity, like encompassing low phonon energy, high chemical and thermal stability, non-hygroscopic nature, and an elevated melting point.^5,17–21^ However, the wide band gap, low electrical conductivity and limited redox-active sites of pristine SMO collectively restrict its rate capability and capacitance retention in SC applications.^5^

To address these drawbacks, cadmium (Cd) has emerged as a promising dopant due to its suitable ionic radius and ability to introduce shallow donor levels, narrow the band gap, enhance conductivity, and create defect sites that promote charge storage and transport.^6^ Although Cd is recognized as a toxic heavy metal, its incorporation at low concentrations within a stable oxide lattice significantly reduces the risk of leaching due to strong chemical bonding and structural confinement. Such controlled doping provides fundamental insight into defect-mediated electrochemical enhancement mechanisms.^22^ Cd doping has been widely studied in various metal oxides, which can reduce charge transfer resistance, create defect sites, and enhance ion diffusion and charge storage.^23^ For instance, Cd-doped-SnO_2_/carbon composites,^23^ simply synthesized Cd–ZnO,^24^ Cd–Co_2_O_3_ fabricated by co-precipitation method,^25^ Cd–Zn co-doped NiO,^26^ and Cd and/or Cu co-doped MIL-68(In)-derived In_2_S_3_,^27^ clearly emphasize the central role of Cd doping in tuning and enhancing the electrochemical properties of materials. However, its use in SMO remains underexplored, offering a promising avenue for future electrode material research as displayed in Tables 1 and S3.

**Table 1:** Comparative analysis of SMO with other doped molybadtes

Doped SrMoO_4_ system	Morphology	Key reported property/application	Reference
Eu^3+^-doped	Irregular particles	Photoluminescence	41
Tb^3+^-doped	Microrods	Green emission	42
Dy^3+^-doped	Platelets	White light emission	43
Fe-doped	Irregular aggregates	Photocatalysis	44
Cd-doped	Uniform nanospheres	Supercapacitor and photocatalysis	Present study

In this study, we therefore report, for the first time, the successful Cd-doping in SMO nanostructures (CSMOs) using a simple and economical co-precipitation method (Fig. 1 and S2). Structural, optical, and electrochemical properties were systematically investigated across different Cd concentrations. The Cd-doped samples exhibited excellent electrochemical performance, showing high specific capacitance and energy density with improved conductivity attributed to reduced charge transfer resistance. Furthermore, the present work introduces a morphology-controlled nanosphere architecture (displayed in Table S1), which facilitates electrolyte diffusion and increases electroactive surface area, leading to improved electrochemical kinetics. An asymmetric supercapacitor device also delivered strong energy and power outputs with outstanding cycling stability, demonstrating its practical potential for energy storage applications.^28–35^

**Fig. 1:**
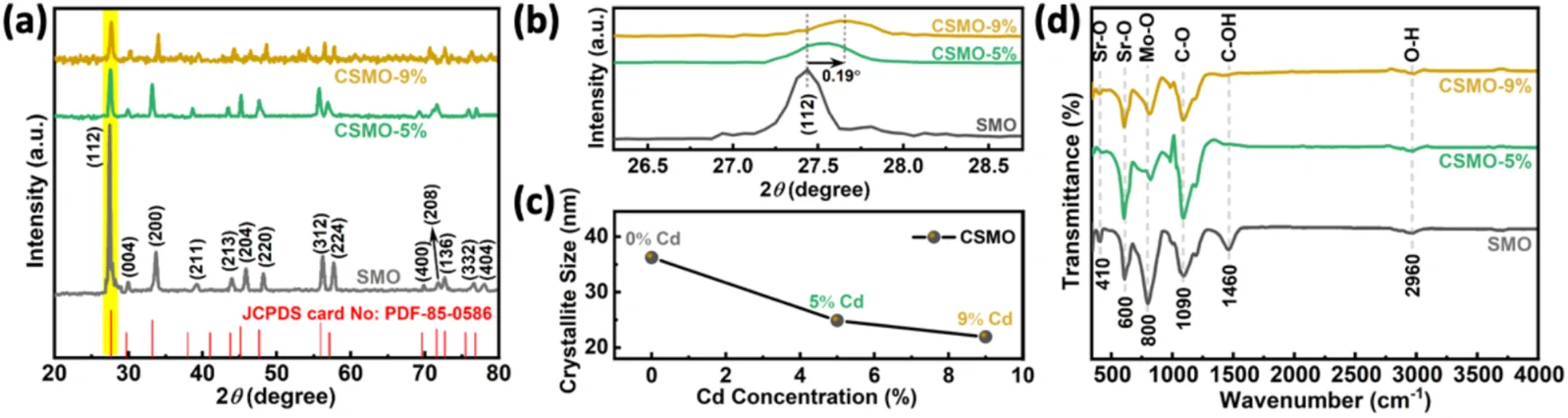
XRD pattern of (a) SMO and Cd-doped CSMO, (b) expanded XRD pattern of (112) plane showing peak shifting, (c) variation in crystallite size with Cd concentration, and (d) FTIR spectra of SMO and Cd-doped CSMO.

Moreover, rising industrial activity has caused severe water pollution by organic contaminants, while photodegradation has emerged as an efficient and sustainable remediation method. Particularly, CSMO-9% demonstrated remarkable photocatalytic performance, achieving nearly complete degradation of Methyl Orange (MO), Methylene Blue (MB), and Rhodamine B (RhB) under UV irradiation, highlighting its potential for effective wastewater treatment applications. These results suggest that CSMOs possess dual functionality, serving effectively as both a high-performance SC electrode material and a potent photocatalyst for environmental remediation.

## Results and discussion

### Structural analysis

#### X-ray diffraction

The structural characteristics of CSMO nanospheres were analyzed using X-ray diffraction (XRD). As revealed in Fig. 1a, standard XRD pattern of SMO crystallize in a tetragonal unit cell (*a* = 5.38 Å, *b* = 12.02 Å; space group *I*4_1_/*a*, JCPDS card no. PDF-85-0586), which closely matches the experimental diffractograms of SMO, CSMO-5%, and CSMO-9%.^5^ The reflections from (112), (004), (200), (211), (213), (204), (220), (312), (224), (400), (208), (136), (332) and (404) planes of SMO lattice correspond to the peaks at 2*θ* values of 27.83°, 29.87°, 33.6°, 39.1°, 43.9°, 45.82°, 48.20°, 55.81°, 57.64°, 69.87°, 71.89°, 72.68°, 76.45°, and 78.0°, respectively.^36^ All the scheelite-type tetragonal structures for CSMO-5% and CSMO-9% are visible through the indexed XRD patterns. A magnified XRD pattern of the peak positioned at 2*θ*_27.43°_ corresponding to the (112) plane displayed in Fig. 1b shows a minor peak shift towards a higher angle for CSMOs, which was due to a lattice contraction resulted from the smaller radius of Cd^2+^ dopant (1.10 Å) than that of Sr^2+^ host (1.18 Å).^37^ Additionally, the reduced intensity diffraction peaks for CSMO-5% and CSMO-9% was because of the induced defects and reduced long-range order through Cd-doping. The resulted lower inter-planer spacing from a higher angle shifting was calculated by Bragg's equation (eqn (1)).1
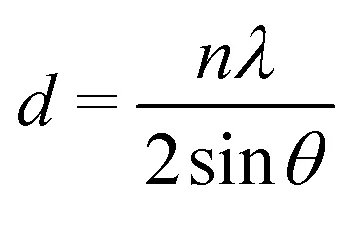
where *n* is an integer, and it is taken to be 1 for discussion convenience. The incident X-rays wavelength is denoted by *λ* and 2*θ* is the angle of diffraction. The calculated *d*-spacing values are consistent with already reported literature and displayed in Table S1.^38^ By employing Debye Scherer's equation (eqn (2)) the standard crystallite sizes of the as-prepared materials were calculated.^36^2
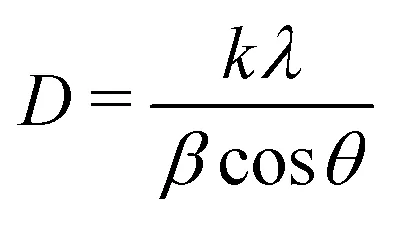
Here, *θ* stands for the Bragg diffraction angle, *β* for the full width at half maximum (FWHM), *k* for the form factor, and *λ* for the wavelength of Cu-Kα radiation (*λ* = 1.5406 Å). The crystallite size of SMO nanospheres was calculated to be 36.23 nm which was decreased to 21.93 nm for CSMO-9% (Fig. 1c), which is consistent with the literature value and reported to enhance the SC performance *via* an increased ion diffusion as well as charge storage.^39,40^

The lattice parameters of pure SMO were found to be *a* = *b* = 0.541 nm and *c* = 1.214 nm, which are consistent with the tetragonal scheelite structure. With Cd doping, a slight decrease in lattice parameters was observed (*a* = *b* ≈ 0.532–0.536 nm, *c* ≈ 1.208 nm), indicating lattice contraction due to substitution of Cd^2+^ ions into the Sr^2+^ sites.

#### FTIR spectroscopy

The functional groups and bonding properties of substances are investigated *via* FTIR analysis. An FTIR spectrum clearly verified the structural development of SMO and CSMOs with the existence of the Sr–O bond (Fig. 1d). FTIR spectrum also suggests the successful incorporation of Cd in the SMO lattice and the existence of a consistent molecular structure in the prepared samples. The distortion of SrO, Mo–O, and Mo–O–Mo bonding is shown by absorption bands that emerged within 400–900 cm^−1^. Previous studies indicate that displacement of MoO bonds in MoO_6_ octahedra or Mo–O–Mo bridges is responsible for IR spectra below 1000 cm^−1^.^36^ The Mo–O anti-symmetric stretch modes of the [MoO_4_] tetrahedron groups are responsible for the broad absorption band seen within 800–900 cm^−1^. Furthermore, Mo–O bending vibrations and Sr–O stretching mode are observable at 800 and 410 cm^−1^, respectively, which were consistent with previously reported literature.^45^ The absorption bands at around 1460 cm^−1^ correspond to C–OH stretching modes.^46^ The wide absorption band at 2960 cm^−1^ indicates O–H stretching modes caused by surface-bound water molecules. The hydroxyl and carboxylic groups in CSMOs may result from surface adsorption of moisture and residual organic species during the synthesis process.^45^ The prominent absorption bands confirm the presence of the scheelite structure in all samples, indicating the potential incorporation of Cd into SMO lattice.

#### Raman spectroscopy

Raman spectroscopy is an efficient method for examining the structural and vibrational properties of nanostructured metal oxides, in which all the samples ranged from 100–1100 cm^−1^ revealed valuable vibrational information as depicted in Fig. 2. Raman band of SMO exhibits characteristic vibrational modes originating from both SrO and MoO_3_ components (Fig. 2a). Peaks associated with SrO and MoO bonds confirmed the presence of structural features from both oxides, consistent with previous literature reports.^47,48^ In molybdates, Raman active vibrational modes are divided into internal and exterior excitation mode groups. Internal vibrations correspond to the [MO_4_]^2−^ molecular group, while external excitation involves the movement of the Sr^2+^ cations. Eqn (3) suggests that there are 26 distinct vibration types present in a tetragonal scheelite primitive cell with a *k* = 0, based on group-theory computations.^49^

3 *Γ* = 3A_g_ + 5E_u_ + 5A_u_ + 5B_g_ + 3B_u_ + 5E_g_

**Fig. 2:**
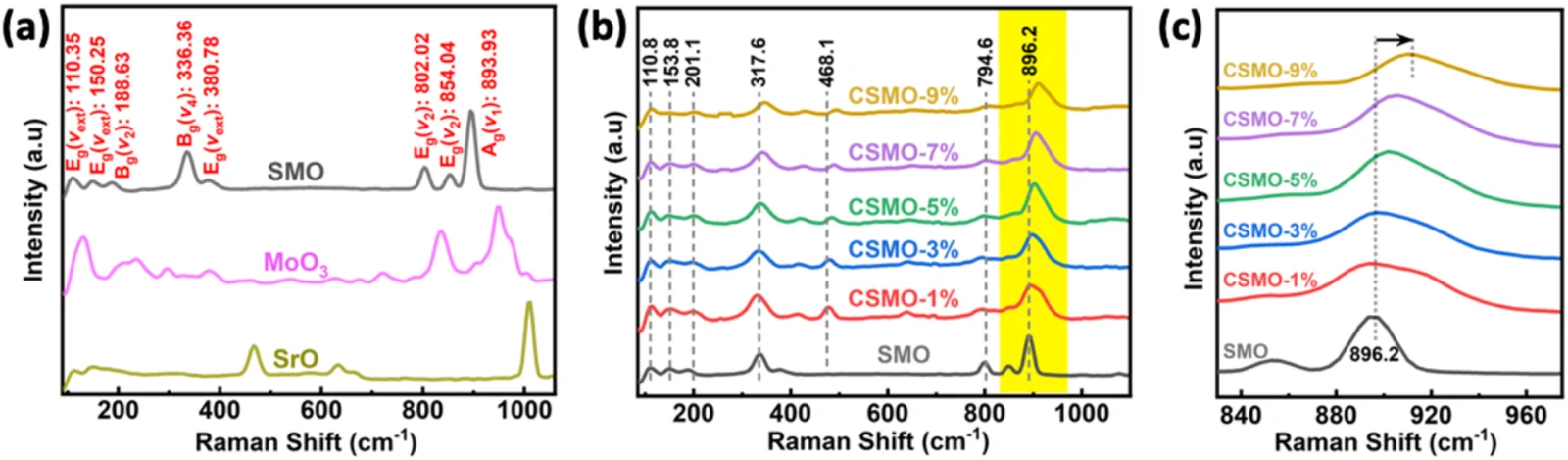
Raman spectra of (a) SMO, MoO_3_, SrO, (b) Cd-doped CSMO, and (c) magnified graph showing peak shift.

While 4A_v_ and 4E_u_ lattice vibrations are particularly dynamic in the infrared frequency region, the excitation modes A_g_, B_g_, and E_g_ may be seen in Raman spectra. In the Raman spectra of SMO and CSMOs illustrated in Fig. 2c, the vibrational modes fall into three categories. The first group detected in the region below 250 cm^−1^ comprises translational (T) and rotational (R) bands linked to connections within Sr^2+^ cations and [MoO_4_]^2−^ tetrahedra [T(E_g_), T(B_g_), R(A_g_) and R(E_g_)].^50^ It is likely that the bending (A_g_, B_g_, and E_g_) and stretching (E_g_ and A_g_) movements of [MoO_4_]^2−^ are the source of the second group of vibrational modes between 250 and 450 cm^−1^ and the third group between 750 and 900 cm^−1^.^51^ The symmetric stretching vibration of [MoO_4_]^2−^ tetrahedral groups is the cause of the Raman active mode detected at 894 cm^−1^. The asymmetric stretching vibrations of *υ*_3_(B_g_) and υ_3_(E_g_), are linked to peaks positioned at 854 and 802 cm^−1^, respectively. Vibrational modes found at 336 cm^−1^ indicate liberty of rotation modes. Furthermore, the vibrational mode at 150 cm^−1^ is the result of exterior excitation modes of Sr^2+^ cations and [MoO_4_]^2−^ tetrahedra. The results correspond with those previously published in the literature.^5^Fig. 2b shows that SMO and CSMOs nanospheres have almost similar patterns, suggesting that the local symmetry and structure are not markedly changed by adding Cd. The peak positions of A_g_ and B_g_ vibration modes show a minor shift towards higher wavenumbers which is shown in Fig. 2c, suggesting Cd dopant effective inclusion into SMO lattice. As discussed earlier in XRD, the difference of ionic radii of Cd^2+^ and Sr^2+^ produces changes in the lattice parameters, such as changes in bond lengths and defect formation which in turn affect the vibrational frequencies of the lattice modes, leading to peak shift. Increasing Cd concentration also reduces the intensity of peaks, likely due to structural variations. These changes can enhance the electrochemical performance of CSMOs, making them a promising candidate for SC electrodes.^52,53^

### Surface morphology and chemical composition

The surface morphology under various magnifications and atomic composition of SMO, CSMO-5%, and CSMO-9% are summarized in Fig. 3. From the perspective of SC applications, improving the ability of the electrode to store energy is facilitated by its surface morphology. The SEM micrographs of SMO and CSMO-5% depicted respectively in Fig. 3a and b show porous, agglomerated, and sponge-like structures made up of interconnected nanosheets or nanoparticles respectively. This interconnected porous network has a large specific surface area, which is ideal for SCs as it allows efficient ionic movement and charge accumulation, leading to better rate capability and power density.

**Fig. 3:**
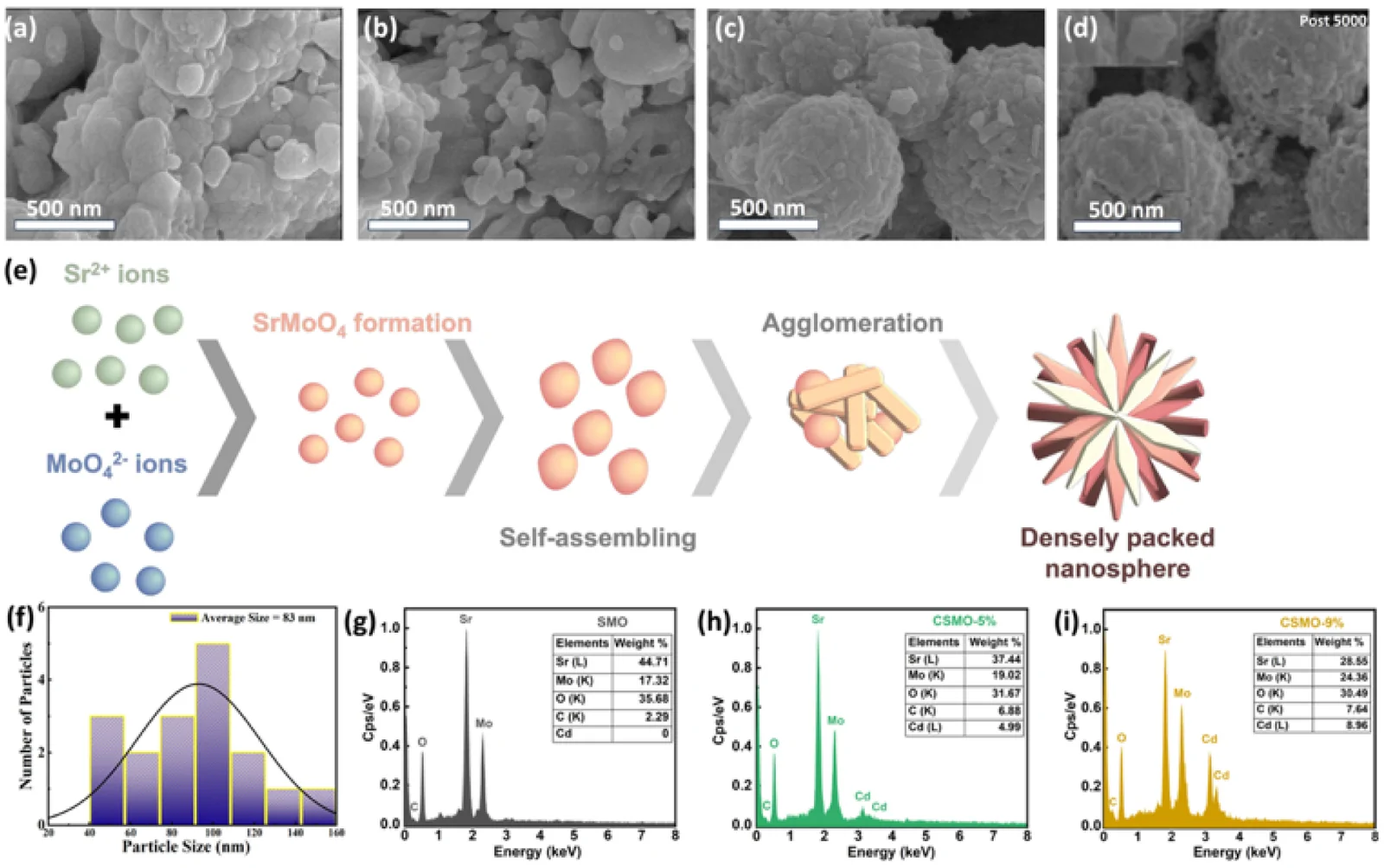
SEM images of (a) SMO, (b) CSMO-5%, (c) CSMO-9%, (d) CSMO-9% after 5000 electrochemical cycles, (e) schematic illustration of morphological development; (f) histogram showing particle size of pure SMO, EDX Spectra of (g) SMO, (h) CSMO-5%, (i) CSMO-9%.

SEM image of CSMO-9% shown in Fig. 3c having a distinct shape of a well-defined nanosphere with densely packed nanosheets and nanoneedles assembled into flower like architectures. Increasing the Cd content in CSMOs promotes a shift from agglomeration to denser, well-defined, and tightly packed spherical morphology (Fig. 3d), suggesting the morphological stability. The distinctive spherical shapes having a large surface area could potentially give advantages in terms of structural stability, electrochemical performance, or specific capacitive behavior. The gaps and voids between the nanosheets and nanoneedles create a porous network, facilitating ion transport and electrolyte penetration, leading to enhanced electrochemical accessibility.^2,54^ The porous surface facilitates electrolyte penetration, enhancing charge transfer during the electrochemical process. Its distinct nanostructure increases active sites and reduces electrode contact resistance.^5^

Particle size analysis from SEM images (Fig. 3f) was performed on pure SMO. The size distribution histogram (Fig. 3f) shows diameters ranging from 20 nm to 140 nm, with a weighted average of 83 nm. The distribution is relatively broad, which is attributed to the wet-chemical synthesis method. Post-cycling morphological characterization was used to assess electrode integrity following long-term riding. As illustrated in Fig. 3d, while some surface roughening and limited particle agglomeration are observed after 5000 cycles, the fundamental electrode architecture remains intact, and no catastrophic failure is seen. This is consistent with the 85% capacitance retention, which remains acceptable for long-term applications. The CSMO nanostructures exhibit high mechanical stability, as evidenced by their 92% capacitance retention.

The EDS results for SMO, CSMO-5%, and CSMO-9% confirm the presence of Sr, Mo, and O as the primary constituents of the host lattice, while the presence of Cd peaks in the doped samples verifies the successful incorporation of Cd into the SMO structure (Fig. 3g–i). A systematic increase in Cd signal intensity from CSMO-5% to CSMO-9% is observed, which is consistent with the increase in dopant concentration. Furthermore, the elemental weight percentages support this trend, showing a gradual increase in Cd content accompanied by corresponding variations in the relative proportions of Sr, Mo, and O. The absence of any additional impurity-related signals indicates that the synthesized samples are compositionally pure within the detection limits of EDS. In addition, the elemental mapping images demonstrate a uniform spatial distribution of all elements across the nanostructures, suggesting homogeneous incorporation of Cd without observable phase segregation or clustering.

### Optical properties

By employing UV-visible spectroscopy, optical properties of the synthesized SMO, SrO, MoO_3_, and CSMOs were thoroughly investigated (Fig. S3). The absorption spectrum of SMO exhibits a characteristic absorption maximum at 252 nm (Fig. S3a), corresponding to electron transitions within the [MoO_4_]^2−^ tetrahedral clusters that are the hallmark of scheelite type structures.^55^ The corresponding Tauc plot reveals a direct band gap of 4.97 eV as a wide and direct band gap semiconductor (Fig. S3b).^56^ In contrast, SrO and MoO_3_ show absorption maxima at 291 nm and 214 nm, with band gaps of 3.03 eV and 4.78 eV, respectively.^57,58^ These findings indicate that SMO possesses a higher band gap than both of its constituent oxides, reflecting a modified electronic structure due to the development of Sr–O, and Mo–O coordination within the lattice.^59,60^ The Wood and Tauc equation (eqn (4)) was used to approximate the optical band gap (*E*_g_) for all prepared samples.

4 *αhυ* = *A*(*E*_g_ − *hυ*)^*n*^

In the given equation, *α* represents the absorption coefficient (calculated as *α* = 2.302 × Abs from absorption spectrum), *E*_g_ denotes the band gap, *A* stands for a constant, and *n* equals 1/2 for indirect and 2 for direct electronic transition. The absorption spectra of SMO and CSMOs (Fig. S3c) show broad shoulder profiles in the UV region (200–400 nm).^60^ SMO and CSMOs exhibit optical absorption spectra affected by direct electronic transitions between the valence bands (VB) and conduction bands (CB), indicating that they are molybdates with a scheelite-type tetragonal crystal structure. This property is ascribed to the molybdenum atoms, which normally form tetrahedral [MoO_4_] clusters in the center of the tetrahedron.^60^ Fig. S3d shows the Tauc plots of SMO and CSMOs, revealing a progressive decline in the direct band gap from 4.96 to 4.09 eV with increasing Cd-dopant concentration. This reduction may be attributed to the introduction of intermediate defect states and lattice strain, which narrow the band gap and promote enhanced electrical conductivity, thus underscores the importance of strategic doping for targeted applications.

### Electrochemical studies

#### Cyclic voltammetry (CV)

CV is an influential technique that is used to investigate the charge storage behavior of electrode materials. CV measurements of SMO and CSMOs were performed at scan rates of 1, 5, 10, 15, 25, 50, and 75 mV s^−1^ within a potential window of 0.0 to 0.5 V in 2 M KOH, as displayed in Fig. 4a–f. The CV curves have distinct redox peaks instead of ideal rectangular forms, showing the presence of faradaic reactions. These characteristics confirm that the charge storage mechanism is neither completely electric double-layer nor purely pseudocapacitive. As a result, the electrode can be categorized as a hybrid system, with a large contribution from battery-type diffusion-controlled reactions, corresponding with the classification described in the literature. The appearance of distinct redox peaks is associated with reversible faradaic reactions involving Sr^2+^ and OH^−^ ions. However, at higher scan rates, the appearance of lower oxidation peaks is attributed to limited ion diffusion, reducing electrochemical activity. The formula given in eqn (5) is used to determine the specific capacitance (*C*_sp_) of the prepared electrode materials from CV plots.5
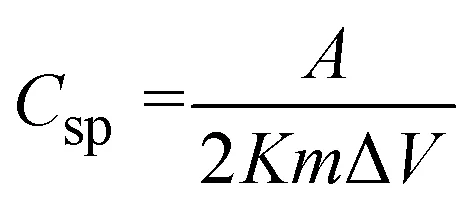
where *m* is the mass, Δ*V* is the window range of potential, *K* is the sweep rate, and *A* is the absolute area under the CV curve.^61^ Sr based surface redox processes in alkaline solutions contributing to charge storage are summarized in (eqn (6)–(9)), involving the formation of Sr(OH)_2_ and SrOOH.^62^ Molybdenum ions enhance conductivity by acting as conductors and without undergoing redox transitions. This improvement boosts specific capacity, consistent with previous studies.^5,63–65^

6 2Sr + O_2_ → 2SrO

7 SrO + OH^−^ → SrOOH + 1e^−^

8 Sr^2+^ + 2OH^−^ → Sr(OH)_2_

9 Sr(OH)_2_ + 2OH^−^ → SrOOH + 1e^−^ + H_2_O

**Fig. 4:**
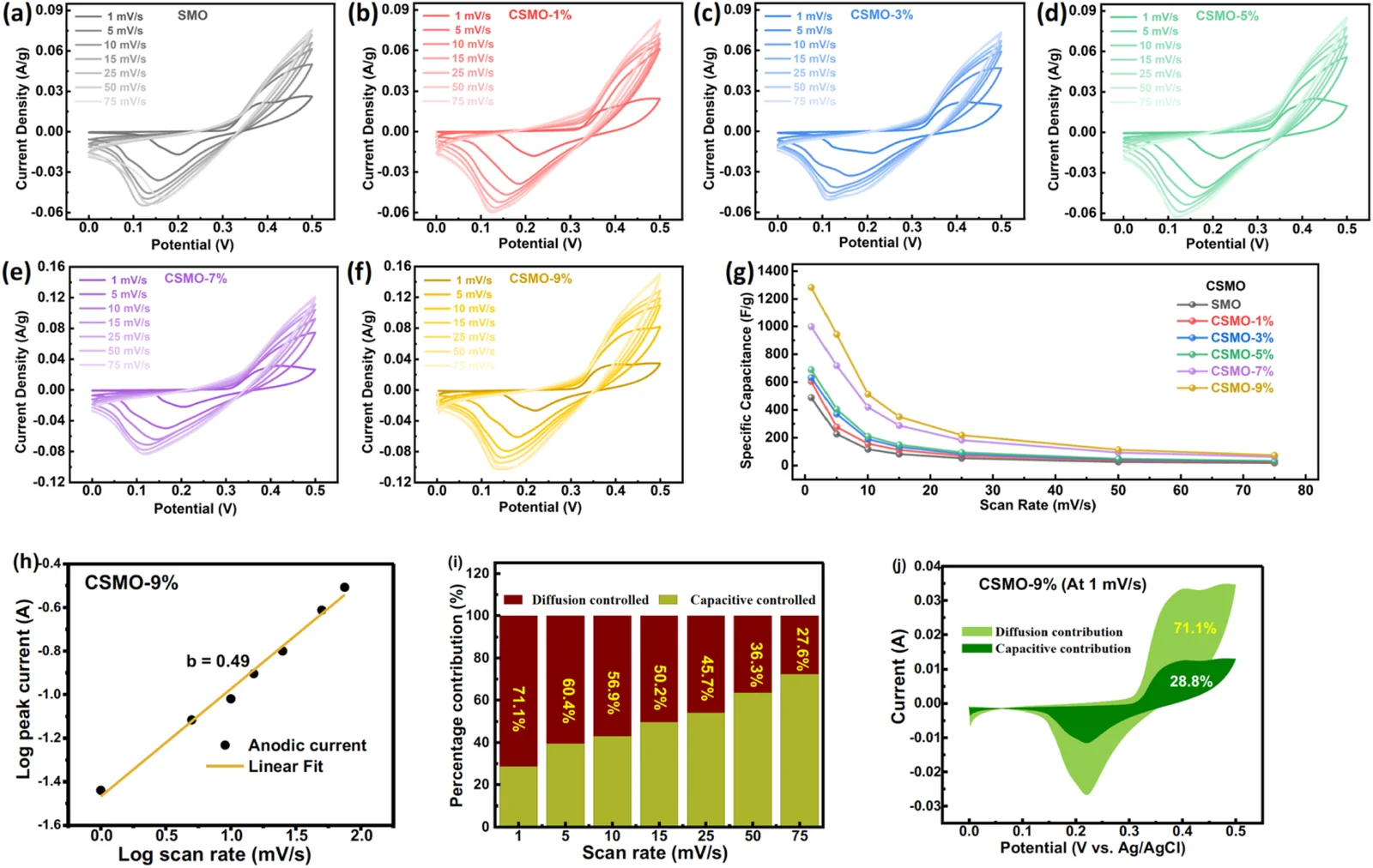
The sweep rate CV of (a) SMO, (b) CSMO-1%, (c) CSMO-3%, (d) CSMO-5%, (e) CSMO-7%, (f) CSMO-9%, and (g) specific capacitance *versus* scan rate of Cd-doped CSMOs, For CSMO-9% (h) *b*-value graph, (i) bar graph of capacitive *vs.* diffusive contribution, (j) separation of capacitive and diffusive currents.

The strong covalent Mo–O bonding and the stable tetrahedral MoO_4_^2−^ structure limit the multivalent redox activity in the prepared CSMO electrodes. Instead, the observed capacitance increase is primarily due to surface-controlled faradaic reactions, defect-induced charge redistribution,^62^ and improved electrolyte accessibility as a result of Cd doping and nanosphere morphology,^66^ which allow for faster charge transfer kinetics than bulk lattice redox processes. Literature has observed similar behaviour in which transition metal molybdates exhibit little bulk Mo redox contribution and dominant surface/defect-controlled capacitance.^66,67^ As displayed in Fig. 4g, *C*_sp_ values decrease gradually with increasing scan rate (Table S2) due to restricted ion access at higher sweep rates. Among all electrodes, CSMO-9% unveiled the high *C*_sp_ values of 1283 F g^−1^ at 1 mV s^−1^, surpassing reported values for related materials (Table S3). This superior performance is attributed to its larger CV area and highlights Cd as a promising dopant for high-performance energy storage materials.

To analyze the further kinetic behavior of the CSMO-9% electrode, Dunn's method and the power law were applied to isolate the capacitive and diffusion-controlled current contribution. As formulated in eqn (10), the total measured current is partitioned into capacitive (*i*_cap_) and diffusion-controlled (*i*_dif_) processes.^68^

10 *i*_total_ = *i*_cap_ + *i*_dif_ = *aν*^*b*^

The kinetic parameters ‘*a*’ and ‘*b*’ were extracted from the log(*i*) *vs.* log(*ν*) plots (*i* = current, *ν* = scan rate). As *b* ≈ 1 implies surface-controlled kinetics, where a *b*-value near 0.5 signifies a predominantly diffusive-controlled process. For the –CSMO-9% electrode, the *b*-value was calculated as 0.49, as presented in Fig. 4h. This suggests that the charge storage is primarily governed by a diffusion-controlled (battery-type) process. The values of capacitive and diffusive currents in voltammteric data at any potential were measured using the modified power law as given below in eqn (11) and (12):

11 *i* = *k*_1_*v* + *k*_2_*v*^1/2^

12 *i*/*v*^1/2^ = *k*_1_*v*^1/2^ + *k*_2_

The values of *k*_1_ and *k*_2_were extracted from the slope and intercept of the linear fit of *i*/*v*^1/2^*versus v*^1/2^, respectively. These constants were utilized in eqn (12) to determine the percentage of each storage mechanism. The resulting Dunn plot (Fig. 4i) reveals the quantification of capacitive and diffusive current percentages, which indicates that at a slow scan rate of 1 mV s^−1^, the diffusive contribution is primary (∼71.1%), while the capacitive contribution accounts for ∼28.8%. This kinetic profile evidenced the dominance of battery-type storage in the CSMo-9% electrode. As the scan rate accelerates to 75 mV s^−1^, the capacitive fraction dominates over the diffusive behavior. This transition in charge storage kinetics is clearly visualized in the fitted CV profiles at 1 mV s^−1^ as presented in Fig. 4j.

#### Galvanostatic charge–discharge (GCD)

GCD measurements were conducted to assess the energy storage capability, rate performance, and electrochemical stability of the fabricated electrodes at various current densities (1, 1.5, 2.5, and 3.5 A g^−1^) within a 0 to 0.5 V potential window in 2 M KOH (Fig. 5a–f). The non-linear and symmetric nature of the GCD curves indicates good reversibility, attributed to faradaic redox reactions. Discharge time decreased with growing current density due to faster ion transport and limited diffusion time at higher rates.^69^

**Fig. 5:**
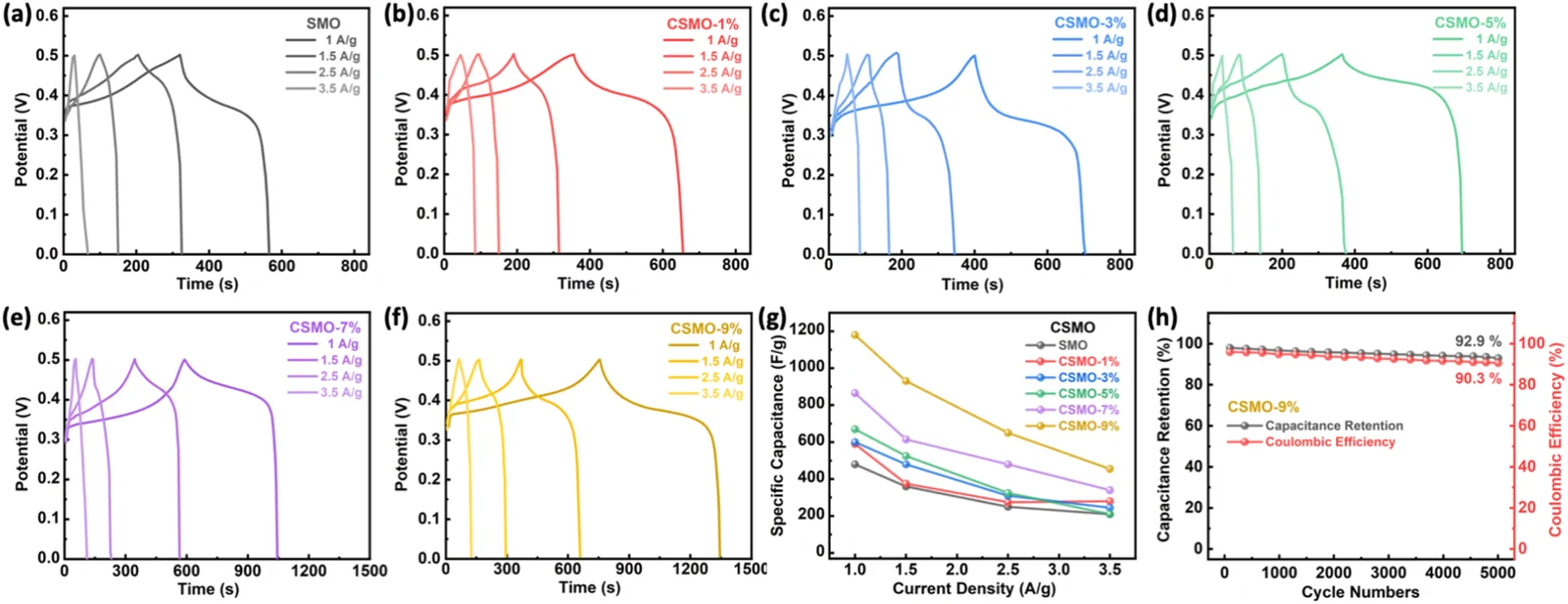
GCD profiles of (a) SMO, (b) CSMO-1%, (c) CSMO-3%, (d) CSMO-5%, (e) CSMO-7%, (f) CSMO-9% at various current densities, (g) variation of specific capacitance as a function of current density, and (h) coulombic efficiency and capacitive retention of CSMO-9%.

We have calculated *C*_sp_ from the discharging time using eqn (13).^70^13
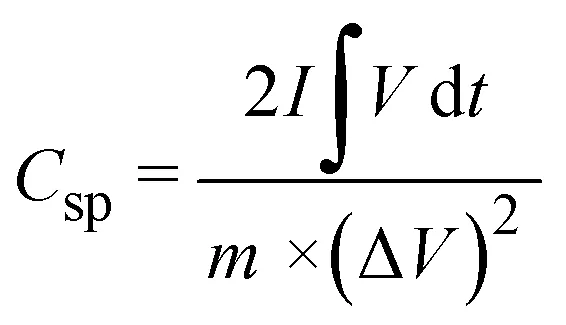


Eqn (14) and (15) were used to compute the energy (*E*_d_) and specific power density (*P*_d_) by utilizing the GCD discharge time.^71^14
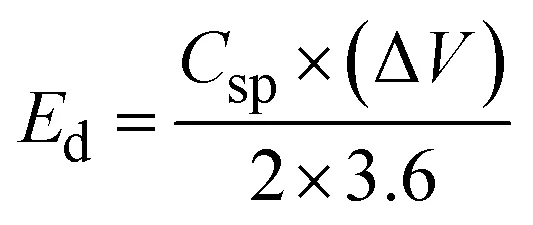
15
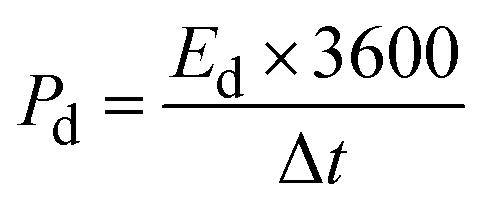
where the discharge window, the discharging current, the active mass, and the discharge duration are successively represented by the Δ*V*, *I*, *m*, and Δ*t*. Among all samples, the CSMO-9% electrode exhibited the highest *C*_sp_ value of 1121 F g^−1^ at 1 A g^−1^ (shown in Fig. 5g, near to the CV-derived value of 1283 F g^−1^. It also achieved a maximum *E*_d_ of 39 W h kg^−1^ in *P*_d_ at 235 W kg^−1^ and, highlighting its suitability for high-performance SC applications (Table S4).

A minimal IR drop at the start of discharge for the CSMO-9% electrode indicates low internal resistance and strong interfacial contact with the electrolyte.^72^ Specific capacitance retention, a key factor for SCs, reflects how well they retain capacity over time. Because it measures the charge transfer efficiency each cycle, coulombic efficiency is a vital metric for appraising electrode performance and stability. Greater reversibility of redox processes and enhanced electrochemical stability are reflected in better coulombic efficiency.

The cycling stability tests in Fig. 5h showed that the CSMO-9% retained 92.9% of its capacitance and 90.3% coulombic efficiency after 5000 electrochemical cycles, outperforming other compositions and literature-reported metal molybdate systems (Table S3). The initial capacitance rise suggests material activation, while the enhanced performance is attributed to the of Cd doping and the intrinsic redox activity of SMO.^5^

#### Electrochemical impedance spectroscopy (EIS)

The EIS analysis was directed to evaluate the charge transport behavior and internal resistance of CSMOs base electrodes in a 2 M KOH electrolyte. Fig. 6 reveals semicircular arcs for all samples, with the arc diameter decreasing from 3% to 9% Cd-dopant loading, indicating reduced charge transfer resistance. The transition from semicircular to linear (Warburg) behavior suggests a shift from charge transfer-controlled to diffusion-controlled processes at lower frequencies. The EIS were modeled by using an equal circuit model comprising uncompensated resistance (*R*_u_) charge-transfer resistance (*R*_ct_), and Warburg resistance (*Z*_w_). In broad terms, the Nyquist plot comprises three segments: low-frequency, medium-frequency, and high-frequency ranges and it provides information regarding the resistance to charge transfer (*R*_ct_), the resistance of the bulk solution (*R*_u_) and the electrode–electrolyte interactions.^73–75^ The Nyquist plot features a straight line at low frequencies due to ion permeation in the electrode and a semicircle at high frequencies from faradaic charge-transfer resistance. The low-frequency region, characterized by a straight line, is associated with *Z*_w_. While the diameter of the semicircle at higher frequencies corresponds to *R*_ct_.^5^

**Fig. 6:**
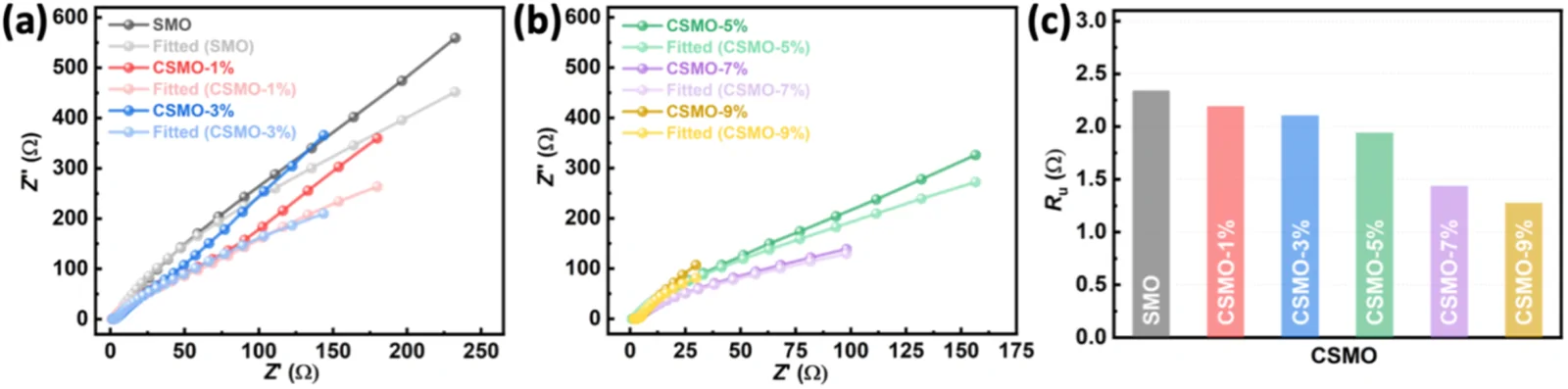
EIS spectra (Nyquist plot) of (a) SMO, CSMO-1%, CSMO-3%, (b) CSMO-5%, CSMO-7%, CSMO-9%, and (c) *R*_u_ values of CSMOs.

The EIS shown in Table S5 suggests that in comparison to SMO, CSMO electrodes have lower values of *Z*_w_, *R*_ct_, and *R*_u_ (Fig. 6c). Particularly, CSMO-9% demonstrated the smallest semicircle and steepest low-frequency slope, signifying improved charge transport kinetics, higher *C*_dl_ and superior energy storage capability. These findings suggest that Cd-dopant effectively improves the electrochemical performance of CSMO, making it a promising candidate for high-performance SCs.

### Asymmetric supercapacitor device (ASCD)

An ASCD based on CSMO-9% nanospheres was systematically evaluated using a two-electrode configuration (having schematic given in Fig. 7a) within a stable potential window of 1.4 V. The positive (active material) and negative (activated carbon) electrodes were fabricated in the same manner as explained in the three-electrode system. Additionally, a Whatman filter paper that had been previously soaked in 2 M KOH electrolyte was placed between two electrodes to design an asymmetric SC device. A Ni foam substrate was coated with a slurry made of CSMO-9% and activated carbon to create one electrode. In order to maintain electrochemical stability and avoid charge mismatch between the electrodes, the device was created using the charge balance concept. Each electrode's stored charge is represented as given in equations (eqn (16)–(18)).^76^

16 *q*^+^ = *q*^−^

17 *m*^+^*C*^+^Δ*V*^+^ = *m*^−^*C*^−^

18
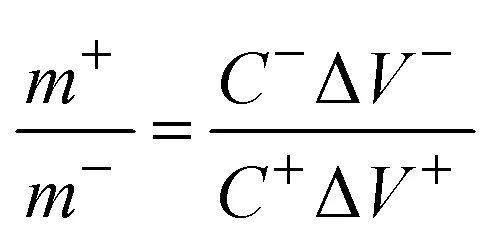
where *m*^+^, *m*^−^, *C*^+^, *C*^−^, Δ*V*^+^ and Δ*V*^−^ correspond to each electrode's mass loading of electrode materials, specific capacitances (F g^−1^), and potential window (V) respectively as established by three-electrode cyclic voltammetry measurements conducted with the same electrolyte. The active material loading was modified in accordance with this charge balance method to guarantee electrochemical equilibrium between the two electrodes. At 1 mV s^−1^ a direct comparison between AC and the AC//CSMO-9% device was carried out (Fig. 7b). The operating potential (OP) for activated carbon was assigned to be −1 to 0 V, while for the active material (CSMO-9%), it was 0–0.5 V. Consequently, the OP for the ASD was extended to 0–1.4 V due to the combined potential windows of AC and CSMO-9%.^77^ This method ensures efficient utilization of both electrodes and prevents charge imbalance during cycling. The electrochemically active CSMO-9% electrode's contribution is highlighted by the device's significantly larger enclosed CV area and stronger current response, which show better charge storage capabilities when compared to AC alone.

**Fig. 7:**
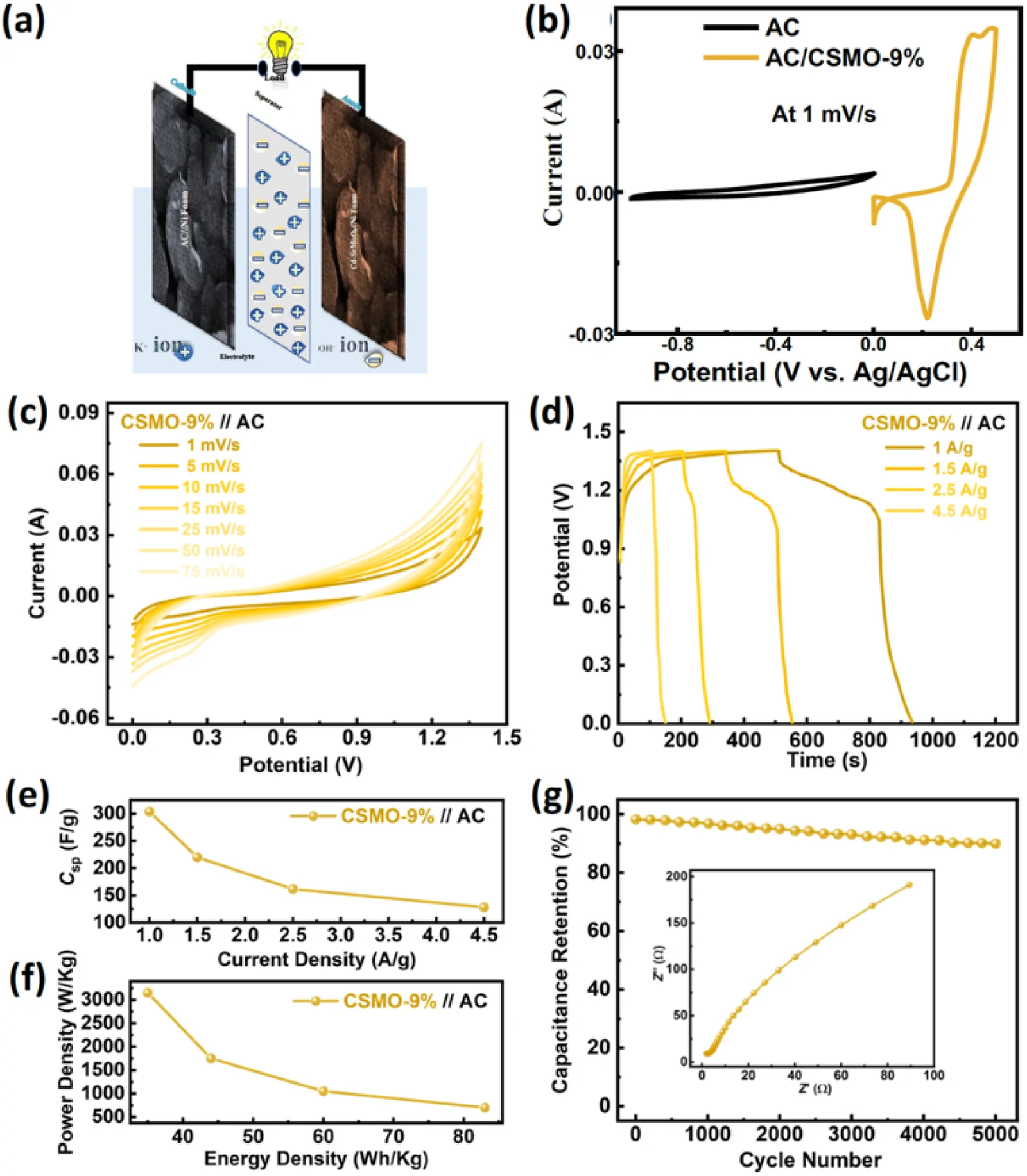
(a) Schematic illustration of ASD mechanism, (b) CV curves of AC and AC//CSMO-9% device at 1 mV s^−1^, electrochemical properties of ASCD consists of CSMO-9%//activated carbon: (c) sweep rate CV, (d) GCD curves at various current densities, (e) variation of specific capacitance as a function of current density, (f) Ragone plot, and (g) capacitive retention (with Nyquist plot as an inset).

In Fig. 7c, the CV curves recorded at various scan rates (1–75 mV s^−1^) exhibit quasi-rectangular profiles with minimal redox peaks, indicating predominant EDLC behavior with slight pseudocapacitive contributions. The well-maintained rectangular profiles across various scan rates (1–75 mV s^−1^) demonstrate excellent rate capability and fast ion kinetics, while symmetric branches and minimal peak distortion at higher scan rates confirm good reversibility and low internal resistance. The *C*_sp_ values calculated from CV data demonstrate a remarkably high value of 352.2 F g^−1^ at a scan rate of 1 mV s^−1^ The GCD curves, obtained at various current densities (1 to 3.5 A g^−1^), display nearly symmetric profiles, confirming good capacitive reversibility (Fig. 7d). The device achieved a high *C*_sp_ value of 304 F g^−1^ at 1 A g^−1^ (Fig. 7e), having *E*_d_ of 84 W h kg^−1^ at *P*_d_ of 700 W kg^−1^. The Ragone plot in Fig. 7f displays the energy and power densities that were computed from GCD profiles. The cycling performance test demonstrates excellent durability, with 90% specific capacitance retention over 5000 continuous cycles as illustrated in Fig. 7g, showing that the manufactured device is electrochemically stable over an extended period. Furthermore, the Nyquist plot in the inset of Fig. 7g sheds light on the resistive properties of device including *R*_ct_ and *R*_u_ values that are calculated to be 1.982 Ω and 0.819 Ω, respectively. These values demonstrate effective ion diffusion and low interfacial resistance. These results establish the potential of CSMO-9% as a promising electrode material for real-world high-performance SC devices. Problem or question you posed in your introduction. Ensure claims are backed up by evidence and explain any complex arguments.

### Photocatalytic evaluation

The prepared SMO and CSMO photocatalysts were subjected to UV light to test their photocatalytic reactivity using three organic dyes (MO, MB, and RhB). In the presence of these catalysts, the optical density curves of MO, MB, and RhB are demonstrated in Fig. 8, S5, and S6, respectively. The photocatalytic performance of SMO, CSMO-5%, and CSMO-9%, was assessed by monitoring MO degradation under UV light irradiation, and then the other two dyes were tested similarly. After 70 minutes, there was essentially no MO degradation in the absence of UV radiation or the as-prepared CSMO catalyst. This finding suggests that self-degradation has a negligible effect. The rate of dye degradation was modeled using a pseudo-first-order kinetic model. The following formulae were used to compute the *k*_app_ and degrading efficiency of the synthesized catalysts (eqn (19) and (20)).^47^19
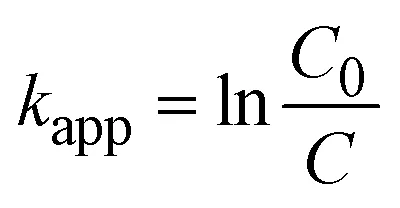
20
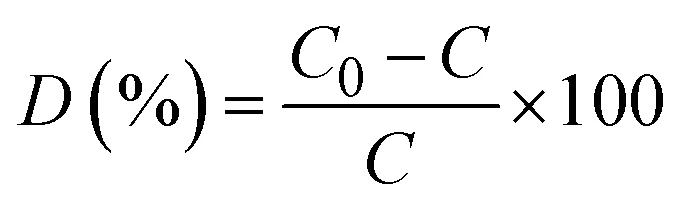


**Fig. 8:**
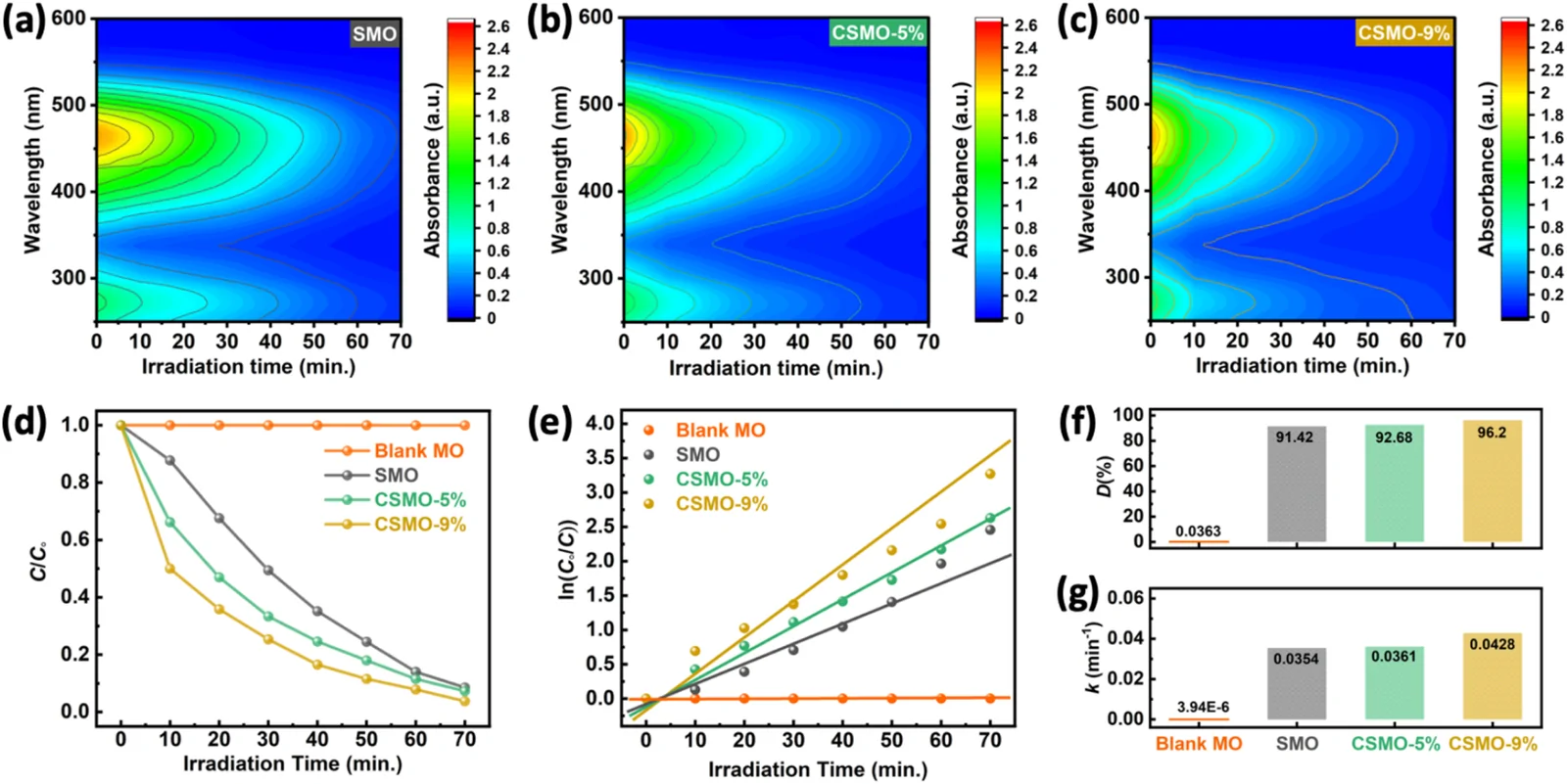
Contour plots of MO dye degradation with (a) SMO, (b) CSMO-5%, (c) CSMO-9%, (d) correlation between *C*/*C*_0_ (e) rate coefficients with kinetic plot against irradiation time, (f) degrading efficiency, and (g) kinetic constants.

In the above equations, *C*_0_, *C*, and *k*_app_ denote the early concentration, concentration at time *t*, and rate constant, respectively. The kinetic behavior of all samples for photodegradation of the dyes is plotted using the pseudo-first-order kinetic model as depicted in Fig. 8d, S5d, and S6d. The photocatalytic breakdown of MO, MB, and RhB, appears to follow a first-order reaction kinetic model based on the linear connection between 
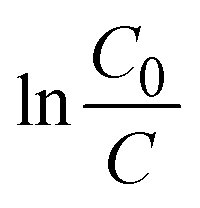
 and time as depicted Fig. 8e, S5e, and S6e, respectively. The slopes of these lines provide the superficial rate coefficients (*k*_app_) of the photocatalyst. The addition of Cd to SMO considerably increased the photocatalytic degradation of the three dyes. CSMO-9% photocatalyst outperformed SMO and CSMO-5% catalysts, reaching significantly higher degradation rates of 96.20% for MO, 95.68% for MB, and 92.48% for RhB, respectively. These findings indicate that Cd-dopant enhances the photocatalytic activity of SMO, possibly due to better charge separation and transport characteristics. One can deduce the following equations (eqn (21)–(24)), as the suggested process for the photocatalytic breakdown of all test dyes for CSMOs.

21 Cd–SrMoO_4_ + *hv* → e^−^ + h^+^

22 h^+^ + H_2_O → ˙OH

23 e^−^ + O_2_ → ˙O^2−^

24 ˙OH + ˙O^2−^ + dye → degradation product

An ideal photocatalyst should have an appropriate band gap, effective charge separation, and high photo-corrosion resistance. A narrow band gap is important for efficient solar spectrum absorption, which is critical for optimal solar energy utilization by the photocatalyst.^78^ UV-visible spectrum summarized in Fig. S3 demonstrate that Cd-dopant decreased the band gap of SMO, which is responsible for the improved photocatalytic abilities of CSMO photocatalysts. When exposed to UV light, CSMO photocatalysts activate and transports electrons (e^−^) from the VB to the CB, by forming holes (h^+^) in the VB. The O_2_ adsorbed on the surface of CSMOs acts as an active site, capturing electrons and generating superoxide radicals (˙O_2_^−^). Furthermore, photo-generated holes in VB can react with water (H_2_O) and hydroxide ions (OH^−^) to produce hydroxyl radicals (˙OH) that operate as potent oxidizing agents. Doping Cd into SMO enhances the separation proficiency of photo-generated charge carriers by trapping electrons on CB with Cd ions, which is beneficial for photocatalytic processes. The CB electrons of CSMO photocatalyst combine with absorbed O_2_ to form O_2_^−^, which can further oxidize the dyes and result in rapid degradation. Meanwhile, the holes in the VB remain on the Cd ions and react with OH^−^ or H_2_O to produce highly oxidizing ˙OH. The dye molecules are immediately oxidized by these radicals into smaller molecules like CO_2_ and H_2_O. Cd-dopant enhances the photocatalytic execution of SMO photocatalyst by prolonging the lifetime of photo-induced electrons and holes, reducing recombination.^79^ The band structure of CSMO-9% photocatalyst showing this activation and its effect on dye degradation is displayed in Fig. S7. CSMO-9% photocatalyst demonstrated superior UV-light photocatalytic activity for dye degradation, explained by greater charge separation, decreased band gap, smaller crystal size, higher surface area, and improved light absorption. These results outperform the results already existing in the literature for SMO and related compounds (Table S6). The faster degradation rates suggest the potential for the use of optimized CSMO photocatalysts in more efficient and sustainable pollutant degradation technologies.

For practical applications, the optimal CSMO-9% photocatalyst was tested for multiple cycles with MB, MO, and RhB dyes under UV-light irradiation, to demonstrate its durability and reusability (Fig. S8). After each use, the catalyst was recovered, cleaned, and dried. Repeated experiments showed minimal performance loss, suggesting a great stability and recyclability for industrial dye degradation processes. Due to the wide band gap (∼4.09 eV), the CSMO-9% photocatalyst is primarily active under UV irradiation. Although this limits its direct application under visible light, future studies may focus on band gap engineering strategies such as heterostructure formation or co-doping to extend its optical response into the visible region.

The excellent dual-functional performance of the optimized CSMO-9% sample in both photocatalytic degradation and supercapacitor applications can be attributed to its tailored electronic structure and enhanced surface redox activity induced by Cd doping.

## Conclusions

This work demonstrates the suitability of CSMOs for both energy storage and photocatalytic applications. The structural, vibrational, and bonding characteristics of the Cd-doped system were systematically analyzed using XRD, Raman, and FTIR, while SEM revealed nanosphere-like morphology for the optimized CSMO-9% sample. The band gap of SMO decreased from 4.96 eV to 4.09 eV after Cd doping, indicating modification in the electronic structure. Electrochemical measurements showed a high specific capacitance of 1283 F g^−1^ at 1 mV s^−1^ and 1180 F g^−1^ at 1 A g^−1^, with good cycling stability (92.9% retention after 5000 cycles). An asymmetric device assembled using CSMO-9% and activated carbon delivered a specific capacitance of 303 F g^−1^ at 1 A g^−1^, along with an energy density of 84 W h kg^−1^ and power density of 700 W kg^−1^, maintaining 90% capacitance retention after 5000 cycles. In addition, photocatalytic studies showed effective degradation efficiencies of 95.68% for MB, 96.20% for MO, and 92.48% for RhB under UV irradiation. These results indicate that CSMO is a promising material for combined electrochemical energy storage and photocatalytic applications.

## Experimental section

### Materials and methods

Sodium molybdate dihydrate (Na_2_MoO_4_·2H_2_O), strontium nitrate (Sr(NO_3_)_2_), sodium hydroxide, cadmium nitrate dihydrate (Cd(NO_3_)_2_·2H_2_O), activated carbon (AC), polyvinylidene difluoride (PVDF), and ethanol (C_2_H_6_O) were purchased from Sigma-Aldrich, while nickel foam substrate was obtained from MTI USA. Pre-purified chemicals and distilled water were used during all experiments.

### Synthesis of SrO and MoO_3_ nanostructures

We employed the co-precipitation technique to synthesize all materials. For the synthesis of strontium oxide nanostructures, we dissolved Sr(NO_3_)_2_ in 50 mL of distilled water for a 0.4 M solution. For the formation of precipitates of strontium hydroxide (Sr(OH)_2_) by maintaining the pH of the solution, we added a sodium hydroxide (NaOH) solution gradually to the agitated strontium nitrate solution. SrO nanospheres were obtained by drying the precipitates at 80 °C and calcination at 500 °C for three hours in the furnace. Similarly, molybdenum oxide nanostructures (MoO_3_ nanospheres) were synthesized by co-precipitation by using nitric acid (HNO_3_) as the precipitating agent instead of NaOH.

### Synthesis of CSMO nanostructures

The samples were fabricated by a simple and surfactant-free co-precipitation synthesis route having a flow chart displayed in Fig. S1. The reaction was conducted at ambient temperature (∼25 °C) under continuous stirring. In the typical synthesis process of SMO, two separate and homogeneous solutions of (Sr(NO_3_)_2_ and Na_2_MoO_4_·2H_2_O precursors were prepared to have a molarity of 0.4 M. After mixing these, the solution was continuously agitated for 60 minutes by a magnetic stirrer the pH of the reaction solution was carefully adjusted to 12 by the dropwise addition of NaOH solution under continuous stirring and maintained throughout the reaction, inducing the formation of white precipitates of SMO, as shown in Fig. S2. After that, the solution was kept for aging for an additional 120 minutes to guarantee complete precipitation and allow the precipitates to settle down. After collecting, these white precipitates were washed away with distilled water and ethanol to eliminate the contaminants through filtration and kept in a drying oven at 80 °C for 24 hours to acquire the dried SMO white powder. For further removal of impurities, the white powder stayed in a muffle furnace for calcination at 450 °C for 3 hours. For the preparation of additional samples with Cd concentrations of 1%, 3%, 5%, 7%, and 9%, the same methodology was employed by using (Cd(NO_3_)_2_·2H_2_O) as the dopant's precursor without any modifications.

The following chemical reactions occur during the synthesis of SrMoO_4_.

25 Na_2_MoO_4_·2H_2_O(aq) + Sr(NO_3_)_2_(aq) → SrMoO_4_(sol) + 2NaNO_3_(aq) + 2H_2_O(l)

### Characterization practices

The crystalline structures of SMO and CSMOs were examined using a JDX-3532 diffractometer from JEOL, Japan, employing Cu-K*α* radiation having 1.5406 Å wavelengths and operational within a 2*θ* range of 20° to 80°. Raman analysis for all of the prepared tasters was made by using a Raman Spectrometer (Model: GMD 532B). The sound structure of the electrode material was examined using a NOVA Nano SEM functioning with 5.0 kV accelerating voltage. EDX examination integrated with the SEM revealed the elemental components of SMO, CSMO-5%, and CSMO-9%. UV-visible absorbance measurements were conducted using a Peak Instrument (Model C7200) spectrometer over the 190–800 nm wavelength range. FTIR analysis was used to verify the molecular conformation of the prepared material in the 400–4000 cm^−1^ range.

By using a mixture of CSMOs (90 wt%) as active material, carbon black (5 wt%) as a conductive material, and PVDF (5 wt%) as binder onto Ni foam (1.5 cm^2^), the working electrodes were fabricated. A homogeneous slurry was prepared using an appropriate amount of ethanol as solvent. Following coating, the electrode was dried at 80 °C for 5 hours to eliminate solvent (Fig. S4). The active mass of the material on Ni foam was estimated to be 1.40 mg cm^−2^. The GAMRY Potentiostat interface 5000 (Model: 14533) electrochemical workstation (Fig. S4) was used to analyze the fabricated electrodes electrochemically by employing CV, EIS, and GCD analysis. A 2 M KOH solution was employed as the electrolyte in a three-electrode assembly with a platinum wire serving as the counter electrode and Ag/AgCl as a reference electrode.

To examine the photocatalytic performance, 25 mg of the photocatalyst was dispersed in a 100 mL glass container containing solutions of test dyes (MO, MB, and RhB) at concentrations of 10 mg L^−1^. The solution was agitated in the dark for 30 minutes to achieve equilibrium between the photocatalyst and the test dyes. The solution was placed 5 cm away from a UV lamp (315–400 nm) and exposed for an additional 70 minutes. Every 10 minutes, 5 mL of dye solution is collected and subjected to centrifugation. The resulting suspension was retained for UV-visible analysis to measure absorbance, indicating the degradation of MO, MB, and RhB over time by observing a reduction in their wavelengths (664.5 nm, 483.5 nm, and 554 nm, respectively) at which they absorb most strongly. After completing the first degradation test, the reaction solution underwent centrifugation to extract the photocatalyst, which was subsequently dried at 60 °C to be used again in next tests.

## Author contributions

Areej Rani, Wasif Ali, Li Chen, and Hui Qi Wong: methodology, investigation, formal analysis, data curation, writing – original draft. Mansour Alhabradi: writing – review and editing, formal analysis. Muhammad Arshad Kamran and Hung-Ju Yen: writing – review and editing, original draft, supervision, resources.

## Conflicts of interest

The authors declare no conflict of interest.

## Supplementary Material

NA-OLF-D6NA00770H-s001

## Data Availability

The data supporting this article have been included as part of the supplementary information (SI). Supplementary information: Comparison of SMO electrodes to previously described electrode materials, calculated *d*-spacing and crystallite size of CSMOs, at various current densities, the calculated specific capacitance, specific power, and specific energy density of CSMOs, the EIS characteristics of all fabricated electrodes based on CSMOs, comparison of photocatalytic performance of CSMO with already reported literature, flowchart of basic steps of Co-precipitation method, schematic illustration of synthesis steps of CSMOs by co-precipitation method, UV-visible absorption spectrum and Tauc plot, schematic illustration of electrode fabrication and experimental set-up of electrochemical workstation, kinetic profiles of the MB dye solution's absorbance as a function of irradiation time in the presence, schematic illustration of the band structure, durability assessment. See DOI: https://doi.org/10.1039/d6na00770h.

## References

[cit1] Pandit B., Johansen M., Andersen B. P., Martínez-Cisneros C. S., Levenfeld B., Ravnsbæk D. B., Varez A. (2023). All-solid-state sodium-ion batteries operating at room temperature based on NASICON-type NaTi2 (PO4) 3 cathode and ceramic NASICON solid electrolyte: A complete in situ synchrotron X-ray study. Chem. Eng. J..

[cit2] Tiwari S. K., Thakur A. K., Adhikari A. D., Zhu Y., Wang N. (2020). Current research of graphene-based nanocomposites and their application for supercapacitors. Nanomaterials.

[cit3] Pandit B., Goda E. S., Ubaidullah M., Shaikh S. F., Nakate U. T., Khedulkar A. P., Kumar D., Doong R.-A. (2022). Hexagonal δ-MnO2 nanoplates as efficient cathode material for potassium-ion batteries. Ceram. Int..

[cit4] Rahmanifar M. S., Hemmati M., Noori A., El-Kady M. F., Mousavi M. F., Kaner R. B. (2019). Asymmetric supercapacitors: an alternative to activated carbon negative electrodes based on earth abundant elements. Mater. Today Energy.

[cit5] Sathiyan A., Rajkumar S., Dhineshkumar S., Merlin J. P. (2024). Electrochemical performance of SrMoO4 as electrode material for energy storage systems. J. Ind. Eng. Chem..

[cit6] Rani B. J., Swathi S., Yuvakkumar R., Ravi G., Rajalakshmi R., Al-Sehemi A. G., Velauthapillai D. (2022). Samarium doped barium molybdate nanostructured candidate for supercapacitors. J. Energy Storage.

[cit7] Mai L.-Q., Yang F., Zhao Y.-L., Xu X., Xu L., Luo Y.-Z. (2011). Hierarchical MnMoO4/CoMoO4 heterostructured nanowires with enhanced supercapacitor performance. Nat. Commun..

[cit8] Yin Z., Chen Y., Zhao Y., Li C., Zhu C., Zhang X. (2015). Hierarchical nanosheet-based CoMoO 4–NiMoO 4 nanotubes for applications in asymmetric supercapacitors and the oxygen evolution reaction. J. Mater. Chem. A.

[cit9] Nti F., Anang D. A., Han J. I. (2018). Facile room temperature synthesis and application of MnMoO4· 0.9 H2O as supercapacitor electrode material. Mater. Lett..

[cit10] Naderi H. R., Ghaderi A., Seyedi Z. S., Eghbali-Arani M. (2019). Facile Synthesis and Characterization of CeMoO 4 Nanostructure via Co-precipitation Method and Investigate its Application Supercapacitor. Anal. Bioanal. Electrochem..

[cit11] Wang Y., He P., Lei W., Dong F., Zhang T. (2014). Novel FeMoO4/graphene composites based electrode materials for supercapacitors. Compos. Sci. Technol..

[cit12] Li P., Ruan C., Xu J., Xie Y. (2019). A high-performance asymmetric supercapacitor electrode based on a three-dimensional ZnMoO 4/CoO nanohybrid on nickel foam. Nanoscale.

[cit13] Ali N. U. H. L., Manoharan S., Pazhamalai P., Kim S.-J. (2022). CuMoO4 nanostructures: a novel bifunctional material for supercapacitor and sensor applications. J. Energy Storage.

[cit14] Seevakan K., Manikandan A., Devendran P., Slimani Y., Baykal A., Alagesan T. (2018). Structural, morphological and magneto-optical properties of CuMoO4 electrochemical nanocatalyst as supercapacitor electrode. Ceram. Int..

[cit15] Seevakan K., Manikandan A., Devendran P., Shameem A., Alagesan T. (2018). Microwave combustion synthesis, magneto-optical and electrochemical properties of NiMoO4 nanoparticles for supercapacitor application. Ceram. Int..

[cit16] Seevakan K., Manikandan A., Devendran P., Slimani Y., Baykal A., Alagesan T. (2019). Structural, magnetic and electrochemical characterizations of Bi2Mo2O9 nanoparticle for supercapacitor application. J. Magn. Magn. Mater..

[cit17] Du Z., Zhao H., Yang C., Shen Y., Yan C., Zhang Y. (2015). Optimization of strontium molybdate based composite anode for solid oxide fuel cells. J. Power Sources.

[cit18] Kogularasu S., Akilarasan M., Chen S.-M., Sheu J.-K. (2021). Scalable and sustainable synthetic assessment between solid-state metathesis and sonochemically derived electrocatalysts (strontium molybdate) for the precise anti-androgen bicalutamide (Casodex™) detection. Microchem. J..

[cit19] Dos Santos D. F., Lovisa L. X., Santiago A. d. A. G., Li M. S., Longo E., Bomio M., Motta F. V. d. (2020). Growth mechanism and vibrational and optical properties of SrMoO 4: Tb 3+, Sm 3+ particles: green–orange tunable color. J. Mater. Sci..

[cit20] Kaewnuam E., Wantana N., Saikaew S., Kaewkhao J. (2020). The luminescence, optical, and structural properties of SrMoO4: Ce 3+ phosphor for photonics material applications. J. Met., Mater. Miner..

[cit21] Paradelas S. M., Gonçalves R. F., Motta F. V., Lima R. C., Li M. S., Longo E., Marques A. P. d. A. (2017). Effects of microwave-assisted hydrothermal treatment and of use of capping reagent on the photophysical properties of SrMoO4 phosphors. J. Lumin..

[cit22] Kaladi Chondath S., Bansal L., Sahu B., Kumar R. (2025). Dopant-induced defect engineering in transition metal oxide/chalcogenide-based electrodes for high-performance supercapacitors: a critical review. ACS Appl. Energy Mater..

[cit23] Dharmaprakash S. (2025). Electrochemical properties of cadmium doped tin oxide film and activated carbon composite electrode. Thin Solid Films.

[cit24] Angelin M. D., Rajkumar S., Ravichandran A., Merlin J. P. (2022). Systematic investigation on the electrochemical performance of Cd-doped ZnO as electrode material for energy storage devices. J. Phys. Chem. Solids.

[cit25] Deng S., Xiao X., Chen G., Wang L., Wang Y. (2016). Cd doped porous Co3O4 nanosheets as electrode material for high performance supercapacitor application. Electrochim. Acta.

[cit26] Ahmed R., Nabi G. (2020). Morphology tailoring and enhanced electrochemical properties of Cd–Zn co-doped NiO nanorods for high performance supercapacitor. Ceram. Int..

[cit27] Farzi M., Moradi M., Hajati S., Toth J., Kazemzadeh A. (2021). Synthesis of rod-like ternary Cu (Cd)-In-S and quaternary Cu-Cd-In-S by controlled ion exchange of MIL-68 (In) derived indium sulfide for high energy-storage capacitor. Synth. Met..

[cit28] Shameem A., Devendran P., Siva V., Murugan A., Sasikumar S., Nallamuthu N., Hussain S., Bahadur S. A. (2020). Robust one-step synthesis of bismuth molybdate nanocomposites: A promising negative electrode for high end ultracapacitors. Solid State Sci..

[cit29] Shameem A., Devendran P., Murugan A., Siva V., Bahadur S. A. (2023). Synergistic effects of lanthanum nanoarchitecture in nickel molybdate nanospheres obtained by single-pot microwave synthesis and their applications in high-end asymmetric devices. J. Phys. Chem. Solids.

[cit30] Bhagwan J., Hussain S. K., Yu J. S. (2019). Facile hydrothermal synthesis and electrochemical properties of CaMoO4 nanoparticles for aqueous asymmetric supercapacitors. ACS Sustainable Chem. Eng..

[cit31] Roshani R., Tadjarodi A. (2021). Synthesis of ZnMoO4 nanofibers by ultrasonic bath method and investigation of their supercapacitive properties. Mater. Today Commun..

[cit32] Veerasubramani G. K., Krishnamoorthy K., Sivaprakasam R., Kim S. J. (2014). Sonochemical synthesis, characterization,
and electrochemical properties of MnMoO4 nanorods for supercapacitor applications. Mater. Chem. Phys..

[cit33] Kashaf A., Kamran M. A., Ullah S., Rani A., Alharbi T., Razzaq A. (2025). Bifunctional vanadium-doped barium molybdate nanostructures for high-performance supercapacitors and efficient photocatalytic degradation. Phys. B.

[cit34] Kumar A., Kumar K. Y., Avadhani D., Prasanna B., Raghu M., Jeon B.-H., Prasana B. (2025). Tailored Yb@ SrMoO_4_/MoS_2_ Hybrid Nanostructures: Application in High-Performance Asymmetric Supercapacitors. Top. Catal..

[cit35] Madhumitha K., Nesaraj A. S. (2026). Facile Synthesis of SrMnO3 Nanorods for High-Capacitance Supercapacitor Devices. J. Indian Chem. Soc..

[cit36] Thongtem T., Kungwankunakorn S., Kuntalue B., Phuruangrat A., Thongtem S. (2010). Luminescence and absorbance of highly crystalline CaMoO4, SrMoO4, CaWO4 and SrWO4 nanoparticles synthesized by co-precipitation method at room temperature. J. Alloys Compd..

[cit37] Wang Y., Xu H., Shao C., Cao J. (2017). Doping induced grain size reduction and photocatalytic performance enhancement of SrMoO4: Bi3+. Appl. Surf. Sci..

[cit38] Liu Y.-F., Dai S.-H., Lu Y.-N., Min H.-H. (2012). Microwave heating synthesis and luminescence property of Eu3+ doped SrMoO4 micro-octahedrons. Powder Technol..

[cit39] Agrawal D. K. (1998). Microwave processing of ceramics. Curr. Opin. Solid State Mater. Sci..

[cit40] Rajkumar S., Subha R., Gowri S., Bella A., Merlin J. P. (2022). Enhanced electrochemical performance of aminophenol-modified ZnO as electrode material for supercapacitors. Ionics.

[cit41] Ranganatha C. L., Palakshamurthy B., Abhilash G., Halappa P., Shivakumara C. (2023). Effect of photoluminescence property in Li+, Ca2+, Bi3+ ions co-doped SrMoO4: Eu3+ phosphors and their Judd–Ofelt analysis for solid-state lighting applications. J. Mater. Sci.: Mater. Electron..

[cit42] Ranganatha C. L., Palakshamurthy B., Abhilash G., Mathew A., Kumar H. S., Shivakumara C. (2023). Development of flexible luminescent films, photoluminescence properties and anti-counterfeiting applications of SrMoO4: Tb3+ green phosphors. J. Mater. Sci.: Mater. Electron..

[cit43] Yogananda H., Yashodha S., Basavaraj R., Bhagya N., Lalithamba H., Sharanakumar T., Kumar C. R., Shetty H. D. (2025). Fabrication of white light emitting SrMoO4: Dy3+ nanophosphors: Photoluminescence, ultrasensitive detection in electrochemical sensing, photocatalytic and advanced forensic applications. Mater. Sci. Eng., B.

[cit44] Luo J., Bai X., Li Q., Yu X., Li C., Wang Z., Wu W., Liang Y., Zhao Z., Liu H. (2019). Band structure engineering of bioinspired Fe doped SrMoO4 for enhanced photocatalytic nitrogen reduction performance. Nano Energy.

[cit45] Marques A. P. A., Tanaka M. T. S., Longo E., Leite E. R., Rosa I. L. V. (2011). The role of the Eu 3+ concentration on the SrMoO 4: Eu phosphor properties: synthesis, characterization and photophysical studies. J. Fluoresc..

[cit46] Yang P., Li C., Wang W., Quan Z., Gai S., Lin J. (2009). Uniform AMoO4: Ln (A= Sr2+, Ba2+; Ln= Eu3+, Tb3+) submicron particles: solvothermal synthesis and luminescent properties. J. Solid State Chem..

[cit47] Ullah S., Kamran M. A., Raheel M., Alharbi T., Ali W., Zou B. (2023). Powering up the energy storage: Exploring the potential of Graphitic Carbon Nitride-Strontium Oxide Nanohybrid for next-generation energy and photocatalytic applications. J. Energy Storage.

[cit48] Windom B. C., Sawyer W., Hahn D. W. (2011). A Raman spectroscopic study of MoS 2 and MoO 3: applications to tribological systems. Tribol. Lett..

[cit49] Basiev T., Sobol A., Voronko Y. K., Zverev P. (2000). Spontaneous Raman spectroscopy of tungstate and molybdate crystals for Raman lasers. Opt. Mater..

[cit50] Sczancoski J., Cavalcante L., Joya M., Varela J. A., Pizani P., Longo E. (2008). SrMoO4 powders processed in microwave-hydrothermal: Synthesis, characterization and optical properties. Chem. Eng. J..

[cit51] Culver S. P., Rabuffetti F. A., Zhou S., Mecklenburg M., Song Y., Melot B. C., Brutchey R. L. (2013). Low-temperature synthesis of AMoO4 (A= Ca, Sr, Ba) scheelite nanocrystals. Chem. Mater..

[cit52] Thambidurai M., Muthukumarasamy N., Agilan S., Sabari Arul N., Murugan N., Balasundaraprabhu R. (2011). Structural and optical characterization of Ni-doped CdS quantum dots. J. Mater. Sci..

[cit53] Murugesan K., Perumal S., Sankaiya A., Lankamsetty K. B., Nagaraju G., Karuppannan B., Rathinam Y., Ganesan R. (2021). Defect induced in 3D-rhombohedral MnCO3 microcrystals by substitution of transition metals for aqueous and solid-state hybrid supercapacitors. ACS Sustainable Chem. Eng..

[cit54] Zhao Y., Zhang X., He J., Zhang L., Xia M., Gao F. (2015). Morphology controlled synthesis of nickel cobalt oxide for supercapacitor application with enhanced cycling stability. Electrochim. Acta.

[cit55] Muralidharan M., Anbarasu V., Elaya Perumal A., Sivakumar K. (2016). Enhanced ferromagnetism in Cr doped SrMoO4 scheelite structured compounds. J. Mater. Sci.: Mater. Electron..

[cit56] Gancheva M., Iordanova R., Avdeev G., Piroeva I., Ivanov P. (2025). Direct mechanochemical synthesis of SrMoO 4: structural and luminescence properties. RSC Mechanochem..

[cit57] Anbu P., Gopinath S. C., Salimi M. N., Letchumanan I., Subramaniam S. (2022). Green synthesized strontium oxide nanoparticles by Elodea canadensis extract and their antibacterial activity. J. Nanostruct. Chem..

[cit58] Toyoda T., Nakanishi H., Endo S., Irie T. (1985). Fundamental absorption edge in the semiconductor CdInGaS4 at high temperatures. J. Phys. D: Appl. Phys..

[cit59] Thongtem T., Phuruangrat A., Thongtem S. (2010). Microwave-assisted synthesis and characterization of SrMoO 4 and SrWO 4 nanocrystals. J. Nanopart. Res..

[cit60] Lei S., Peng X., Li X., Liang Z., Yang Y., Cheng B., Xiao Y., Zhou L. (2011). Controlled fabrication of SrMoO4 hierarchical nanosheets in a surfactant-assisted nonaqueous system. Mater. Res. Bull..

[cit61] Kamran M. A., Siddique S., Ullah S., Alharbi T., Raza M., Usama M., Zou B. (2023). Bifunctional strontium-doped barium oxide nanorods as promising photocatalysts and electrodes for energy storage applications. J. Energy Storage.

[cit62] Goda E. S., Hong S. E., Pandit B., ur Rehman A., Singu B. S., Akhtar M. S., Sohail M., El-Bahy S. M., Gamal H., Yoon K. R. (2023). Metal-organic framework (MOF) templated hierarchical Al-doped CoxP@ graphene composite: a promising solid-state asymmetric supercapacitor with PANI derived carbon nanorods. J. Alloys Compd..

[cit63] Seevakan K., Manikandan A., Devendran P., Baykal A., Alagesan T. (2018). Electrochemical and magneto-optical properties of cobalt molybdate nano-catalyst as high-performance supercapacitor. Ceram. Int..

[cit64] Shameem A., Devendran P., Siva V., Packiaraj R., Nallamuthu N., Asath Bahadur S. (2019). Electrochemical performance and optimization of α-NiMoO 4 by different facile synthetic approach for supercapacitor application. J. Mater. Sci.: Mater. Electron..

[cit65] Zhu Z., Sun Y., Li C., Yang C., Li L., Zhu J., Chou S., Wang M., Wang D., Li Y. (2021). Mini-review: progress on micro/nanoscale MnMoO 4 as an electrode material for advanced supercapacitor applications. Mater. Chem. Front..

[cit66] Khedulkar A. P., Yu W.-J., Pandit B., Doong R.-A. (2024). Boosting supercapacitor performance with a cobalt hydroxide in-situ preparation orange peel biochar flower-like composite. J. Energy Storage.

[cit67] Sharma P., Minakshi M., Whale J., Jean-Fulcrand A., Garnweitner G. (2021). Effect of the anionic counterpart: Molybdate vs. tungstate in energy storage for pseudo-capacitor applications. Nanomaterials.

[cit68] Goda E. S., Pandit B., Hong S. E., Singu B. S., Kim S. K., Moustafa E. B., Yoon K. R. (2022). Zeolitic imidazolate framework-67 derived Al-Co-S hierarchical sheets bridged by nitrogen-doped graphene: Incorporation of PANI derived carbon nanorods for solid-state asymmetric supercapacitors. J. Energy Chem..

[cit69] Meher S. K., Justin P., Rao G. R. (2010). Pine-cone morphology and pseudocapacitive behavior of nanoporous nickel oxide. Electrochim. Acta.

[cit70] Pandit B., Sankapal B. R. (2022). Cerium selenide nanopebble/multiwalled carbon nanotube composite electrodes for solid-state symmetric supercapacitors. ACS Appl. Nano Mater..

[cit71] Dong X., Cao Y., Wang J., Chan-Park M. B., Wang L., Huang W., Chen P. (2012). Hybrid structure of zinc oxide nanorods and three dimensional graphene foam for supercapacitor and electrochemical sensor applications. RSC Adv..

[cit72] Kumar R. D., Karuppuchamy S. (2014). Microwave-assisted synthesis of copper tungstate nanopowder for supercapacitor applications. Ceram. Int..

[cit73] Shayeh J. S., Ehsani A., Nikkar A., Norouzi P., Ganjali M. R., Wojdyla M. (2015). Physioelectrochemical investigation of the supercapacitive performance of a ternary nanocomposite by common electrochemical methods and fast Fourier transform voltammetry. New J. Chem..

[cit74] Leng Y., Ge S., Marple D., Yang X.-G., Bauer C., Lamp P., Wang C.-Y. (2017). Electrochemical cycle-life characterization of high energy lithium-ion cells with thick Li (Ni0. 6Mn0. 2Co0. 2) O2 and graphite electrodes. J. Electrochem. Soc..

[cit75] Kamran M. A., Ali W., Alharbi T. (2024). Facile synthesis of novel cobalt molybdenum sulfide-graphitic carbon nitride (CoMoS4/g-C3N4) nanocomposite electrode for improved supercapacitor and photocatalytic applications. J. Energy Storage.

[cit76] Redondo E., Le Fevre L. W., Fields R., Todd R., Forsyth A. J., Dryfe R. A. (2020). Enhancing supercapacitor energy density by mass-balancing of graphene composite electrodes. Electrochim. Acta.

[cit77] Ali S., Hassan H., Iqbal M. W., Afzal A. M., Amin M. A., Alhadrami A., Alqarni N. D., Umar E. (2023). Analyzing synthesis routes for BaCuPO 4: implications for hydrogen evolution and supercapattery performance. RSC Adv..

[cit78] Bai X., Wang L., Zhu Y. (2012). Visible photocatalytic activity enhancement of ZnWO4 by graphene hybridization. ACS Catal..

[cit79] Zhang L., Zheng G., Dai Z., Zhao X. (2019). Synthesis of Co doping SrMoO4 for enhanced photocatalytic performance via hydrothermal method. Dig. J. Nanomater. Biostruct..

