# Supplementary material for: Cadmium-doped molybdenum nanospheres as emerging energy and environmental electrode materials
Source: Nanoscale Adv. 2026 Oct 1. Online ahead of print. doi: 10.1039/d6na00770h (PMC13643389; doi:10.1039/d6na00770h)
Supplement: Supplementary file 1 [file NA-OLF-D6NA00770H-s001.pdf]

Supplementary Information (SI) for Nanoscale Advances.
This journal is © The Royal Society of Chemistry

Supporting Information

Cadmium-doped Molybdenum Nanospheres as Emerging Energy and Environmental Electrode Materials

Muhammad Arshad Kamran,[1,*] Areej Rani,[1] Wasif Ali,[2] Mansour Alhabradi,[3,*] Li Chen,[2] Hui Qi Wong[2] and Hung-Ju Yen[2,*]

[1] *Department of Physics, University of Okara, Okara, Pakistan.*
[2] *Institute of Chemistry, Academia Sinica, Taipei 11529, Taiwan.*
[3] *Department of Physics, College of Science, Majmaah University, Majmaah, Saudi Arabia.*

Email address: m.kamran@uo.edu.pk (M.A.K.); M.alhabradi@mu.edu.sa (M.A.); hjyen@gate.sinica.edu.tw (H.J.Y.)

Table S1. Calculated crystallite size and *d*-spacing of SMO and CSMOs.

Sample Name	FWHM [degree]	Peak Position [$2\theta°$]	Crystallite Size (*D*) [nm]	Interplanar distance (*d*) [nm]
**SMO**	0.2358	27.4290	36.24	0.3246
**CSMO-5%**	0.3436	27.5689	24.87	0.3235
**CSMO-9%**	0.3898	27.6271	21.93	0.3230

Table S2. Specific capacitance values of the prepared electrodes at different sweep rates.

Electrode Material	75 mV/s [F/g]	50 mV/s [F/g]	25 mV/s [F/g]	15 mV/s [F/g]	10 mV/s [F/g]	5 mV/s [F/g]	1 mV/s [F/g]
**SMO**	19	26	53	83	117	227	488
**CSMO-1%**	24	37	70	112	156	277	604
**CSMO-3%**	31	44	83	134	190	370	632
**CSMO-5%**	32	49	94	150	210	405	689
**CSMO-7%**	62	93	183	287	420	718	998
**CSMO-9%**	74	114	218	350	513	942	1283

Table S3. Comparison of CSMO electrodes to previously described electrode materials.

Electrode Material	Electrolyte	Current Density (or) scan rate	Specific Capacitance [F/g]	Retention (No. of Cycles)	References
**$NiMoO_4$**	1 M KOH	2 mA/cm^2	450	94 % (1000)	S1
**$CuMoO_4$**	1 M KOH	1 mA/cm^2	127	82.5 % (1000)	S2
**$Bi_2Mo_2O_9$**	3 M KOH	0.4 A/g	197	---	S3
**$CoMoO_4$**	1 M KOH	1 mA/cm^2	133	---	S4
**α-$NiMoO_4$**	2 M KOH	5 mV/s	444	98.6 % (1000)	S5
**Ln:$BiMoO_6$**	3 M KOH	5 mV/s	662.8	87 % (2000)	S6
**Ln-$NiMoO_4$**	3 M KOH	1 mA/cm^2	1264.64	98.86 % (5000)	S7
**$CaMoO_4$**	1 M KOH	2 A/g	118	85 % (5000)	S8
**$ZnMoO_4$**	3 M KOH	2 mV/s	487	---	S9
**α-$MnMoO_4$**	2 M NaOH	0.5 mA/cm^2	168.32	96 % (2000)	S10
**$MnMoO_4$/ $CoMoO_4$**	2 M NaOH	1 A/g	187.1	---	S11
**Sm-$BaMoO_4$**	1 M KOH	1 A/g	135	83.38 % (5000)	S12
**Cd-ZnO**	---	1 A/g	627	93.3 % (5000)	S13
**Cd-Co_3O_4**	6 M KOH	1 A/g	737	96 % (1000)	S14
**Cd-Cu co-doped In_2S_3**	---	2 A/g	402	---	S15
**Cd-Zn co-doped NiO**	2 M NaOH	5 mV/s	1485.19	82.8 % (---)	S16
**$SrMoO_4$**	1 M KOH	5 mV/s	384	91 % (5000)	S17
**CSMO**	2 M KOH	1 mV/s	1283	92.9 % (5000)	Present Work

Table S4. At various current densities, the calculated specific capacitance, specific power, and specific energy density of SMO and CSMOs.

Electrode Material	Discharge Time [s]	Current Density [A/g]	Specific Capacitance [F/g]	Energy Density [Wh/kg]	Power Density [W/kg]
**SMO**	240<br>120<br>50<br>30	1<br>1.5<br>2.5<br>3.5	480<br>360<br>250<br>210	17<br>13<br>9<br>7	250<br>375<br>625<br>875
**CSMO-1%**	295<br>125<br>55<br>40	1<br>1.5<br>2.5<br>3.5	590<br>375<br>275<br>280	20<br>13<br>9<br>8	250<br>375<br>625<br>875
**CSMO-3%**	300<br>160<br>62<br>35	1<br>1.5<br>2.5<br>3.5	600<br>480<br>310<br>245	21<br>17<br>11<br>9	250<br>375<br>625<br>875
**CSMO-5%**	335<br>175<br>65<br>30	1<br>1.5<br>2.5<br>3.5	670<br>525<br>325<br>210	23<br>18<br>11<br>7	250<br>375<br>625<br>875
**CSMO-7%**	455<br>220<br>105<br>55	1<br>1.5<br>2.5<br>3.5	910<br>660<br>525<br>385	32<br>23<br>18<br>13	250<br>375<br>625<br>875
**CSMO-9%**	590<br>310<br>130<br>65	1<br>1.5<br>2.5<br>3.5	1180<br>930<br>650<br>455	41<br>32<br>22<br>16	250<br>375<br>625<br>875

Some parameters are used in the above table; discharging time (Δt), current density (I_{m}), specific capacitance (C_{sp}), specific energy (E_{d}), and power density (P_{d})) sequentially.

Table S5. The EIS characteristics of all fabricated electrodes based on CSMOs.

Electrode Material	R_u [ohm]	C_{dl} [10^{-3} F]	Z_w [10^{-3} ohm]	R_{ct} [10^{-3} ohm]
**SMO**	2.341	0.6824	4.701	17.18
**CSMO-1%**	2.172	0.7405	3.719	9.661
**CSMO-3%**	2.107	0.7941	3.646	5.735
**CSMO-5%**	1.942	0.8233	3.41	5.073
**CSMO-7%**	1.337	1.086	2.922	2.99
**CSMO-9%**	1.276	1.618	2.021	2.955

Table S6. Comparison of photocatalytic performance of CSMOs with reported literature.

Photocatalyst	Dye	Light Source	Degradation Time [min.]	Photocatalytic Efficiency (*D*) [%]	Reference
**CSMO-9%**	MO	UV light	70	96.20	Present work
	MB			95.68	
	RhB			92.48	
**$SrMoO_4$**	MB	Visible	120	50	S18
**$BaMoO_4$**	MB	UV light	40	>85	S19
**$BaMoO_4$**	MO	UV light	70	75	S20
**$CaMoO_4$**	MO	UV light	60	70	S21
**$BaMoO_4$: Er^{3+}/Yb^{3+}**	RhB	Solar light	90	70.66	S22
**Ce-$SrMoO_4$**	MB	UV light	150	95.5	S23
**Co-$SrMoO_4$**	MB	Visible	120	90.8	S24
**Bi-$CdMoO_4$**	RhB	UV light	180	85	S25
**Ce-$CaMoO_4$**	MB	UV light	120	96.62	S26

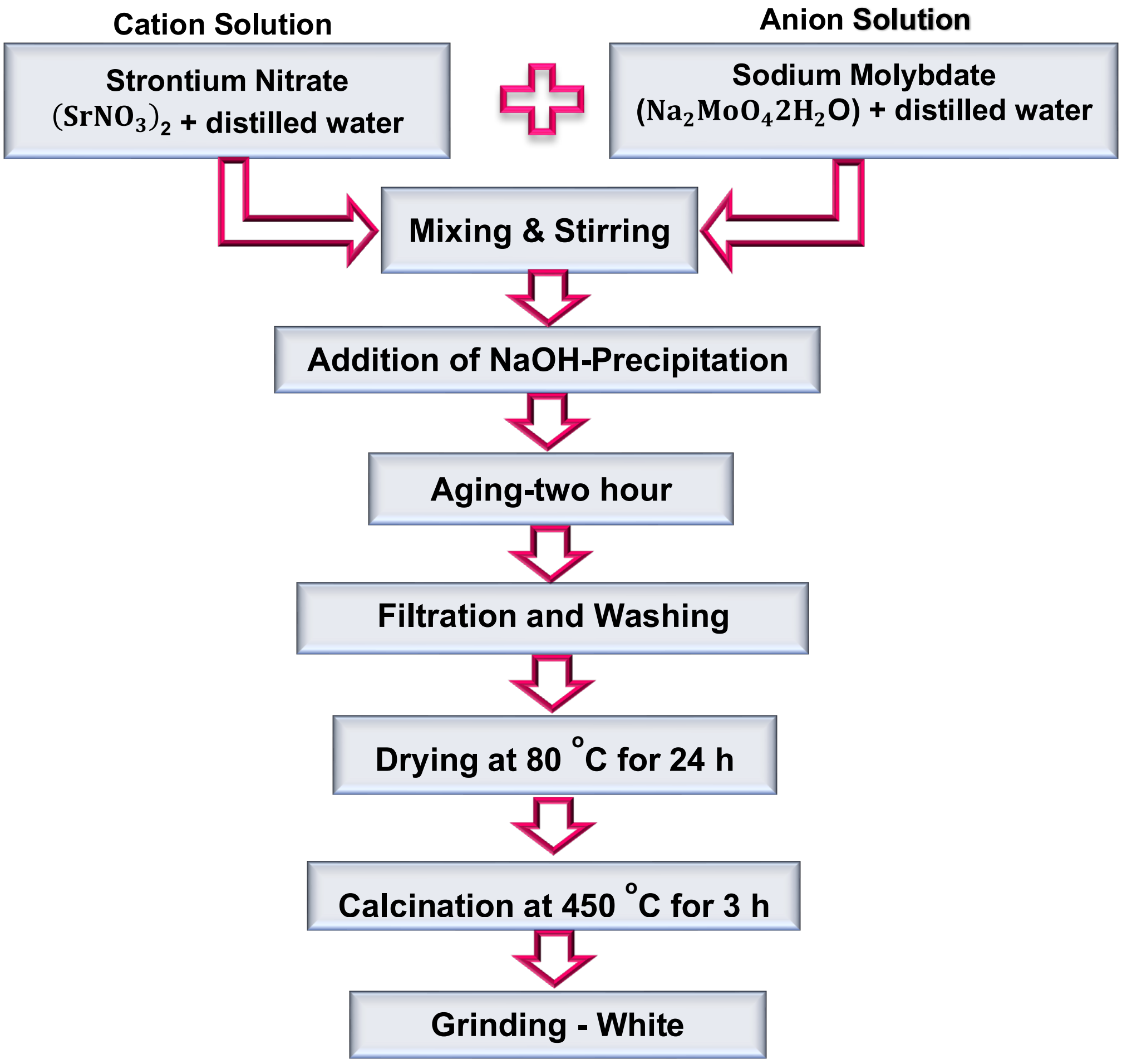

Figure S1. Flowchart of basic steps of Co-precipitation method.

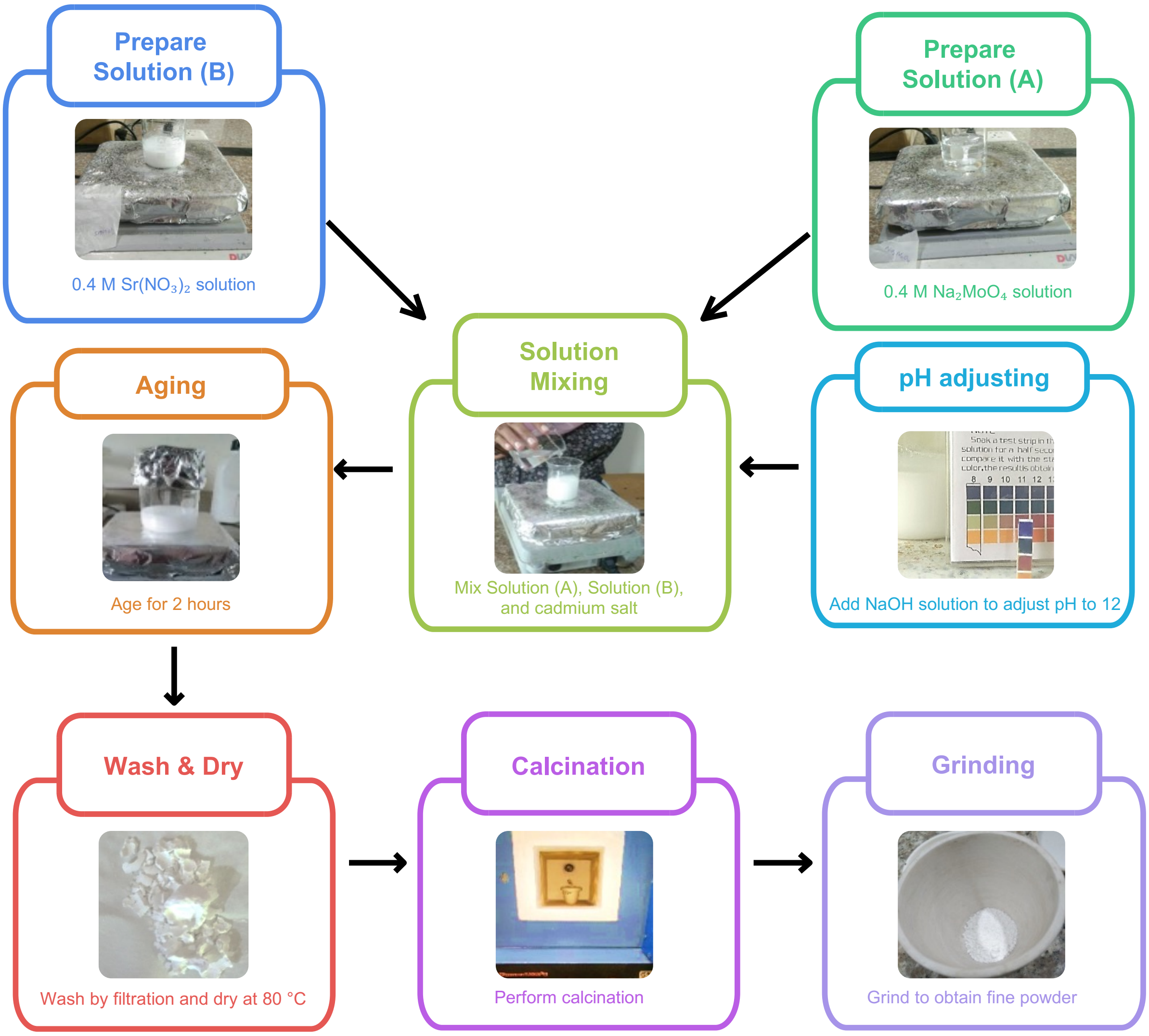

Figure S2. Schematic Illustration of synthesis steps of CSMOs by Co-precipitation method.

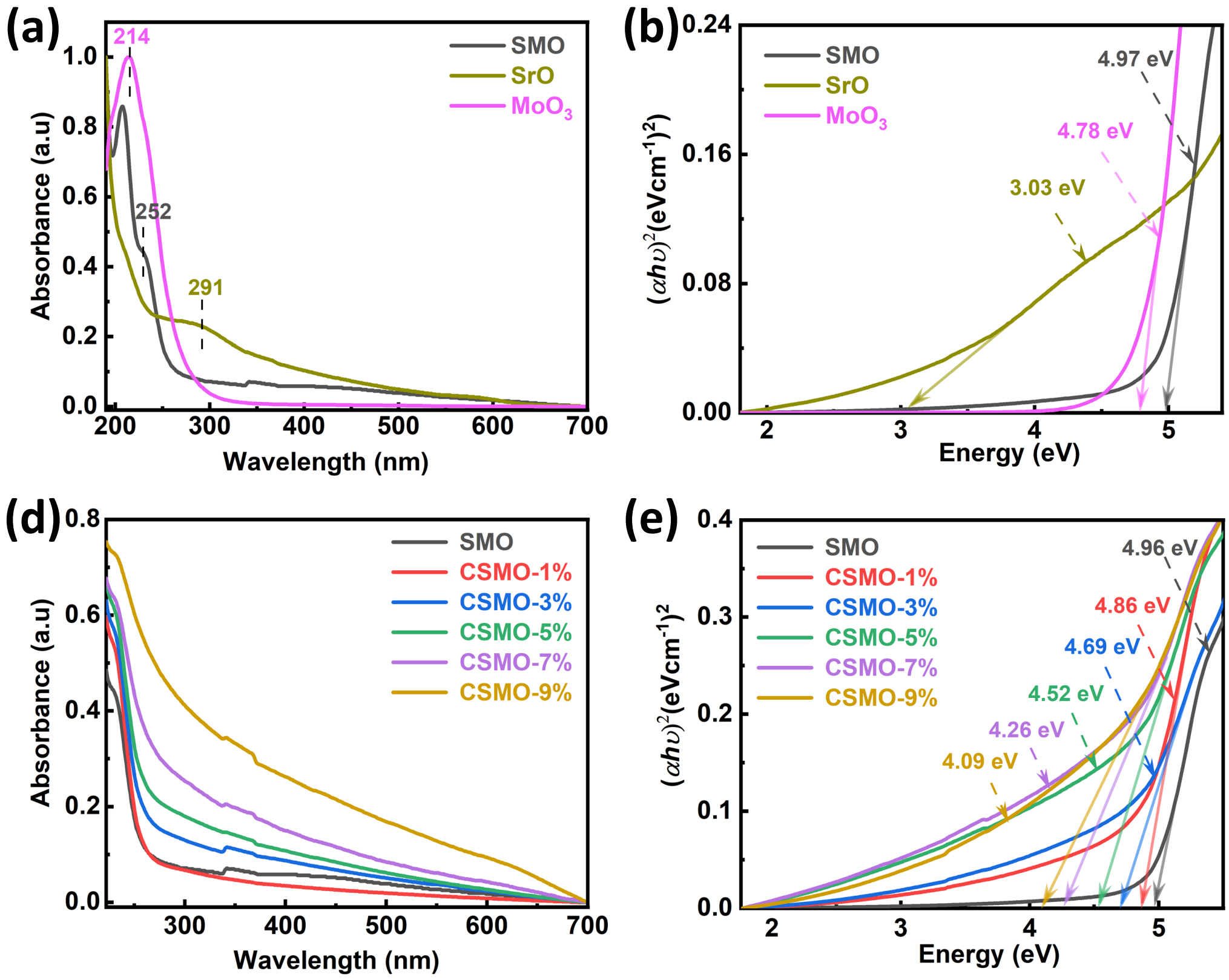

Figure S3. (a) UV-Visible absorption spectrum and (b) Tauc plot of SrO, MoO_3, SMO; (c) UV-Visible absorption spectrum and (d) Tauc plot of SMO and CSMOs.

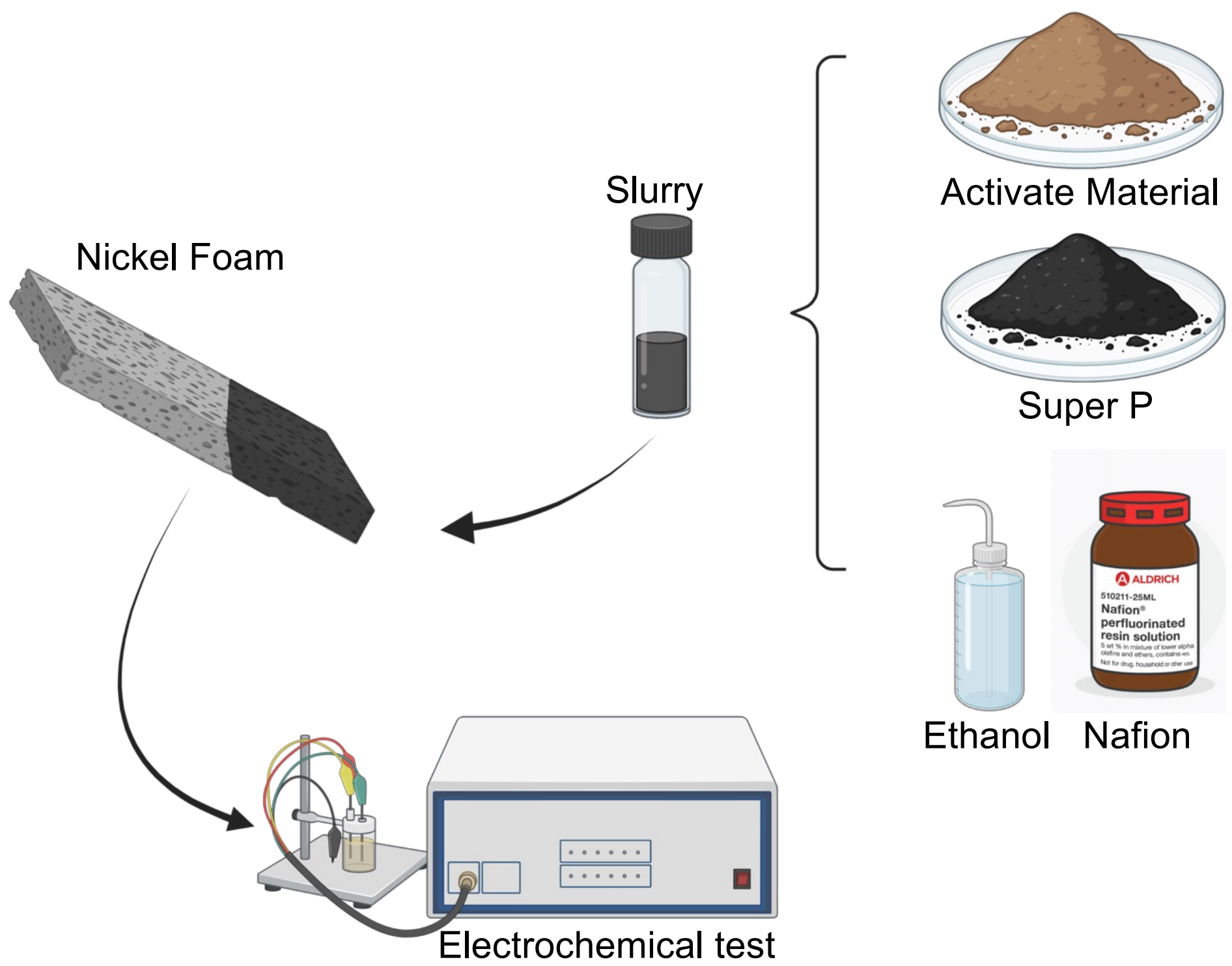

Figure S4. Schematic illustration of Electrode Fabrication and experimental set-up of Electrochemical Workstation at MRL, University of Okara.

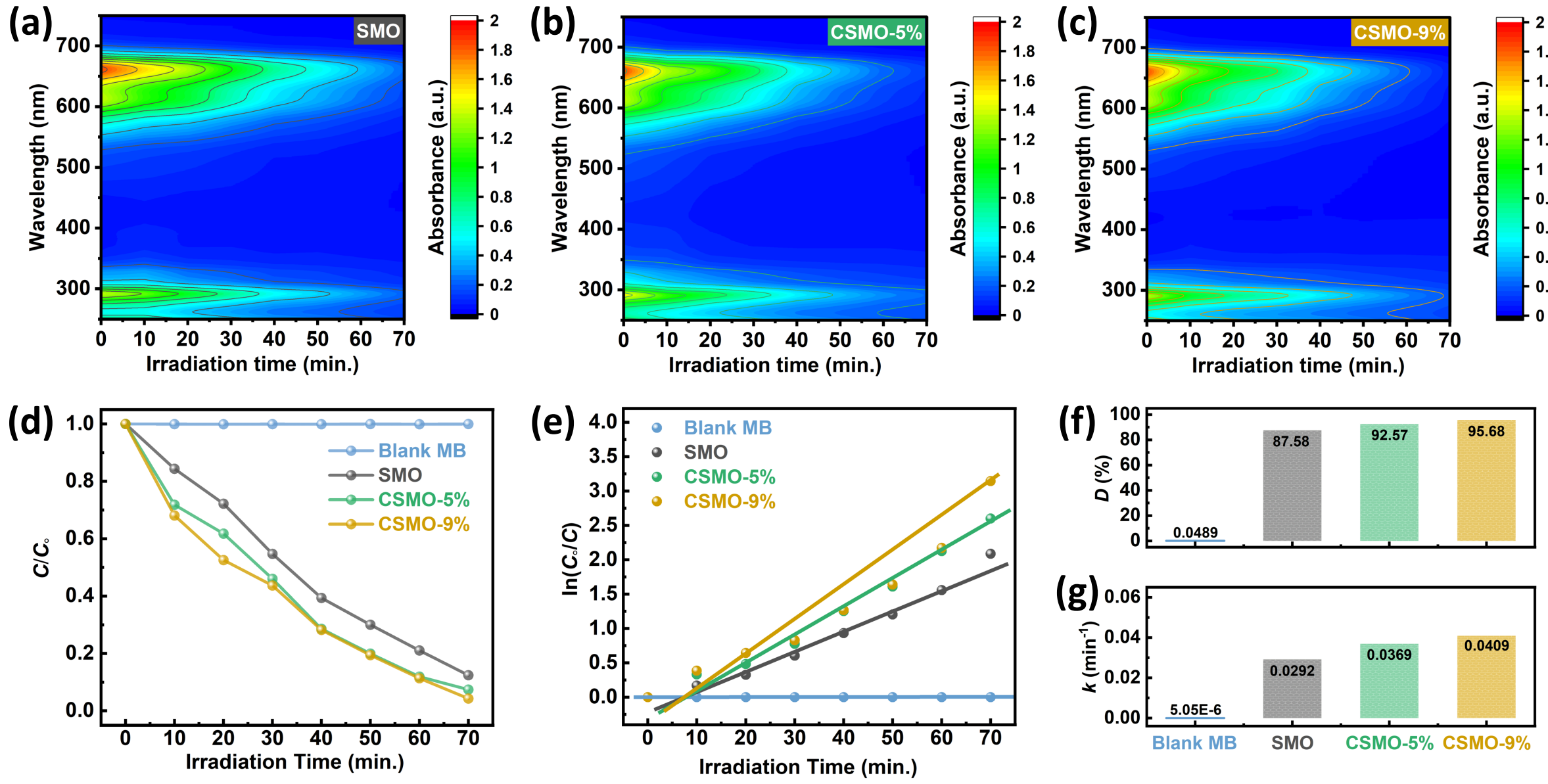

Figure S5. Contour plots of MB dye degradation with (a) SMO, (b) CSMO-5%, (c) CSMO-9%, (d) correlation between C/C_o (e) rate coefficients with kinetic plot against irradiation time, (f) degrading efficiency, and (g) kinetic constants.

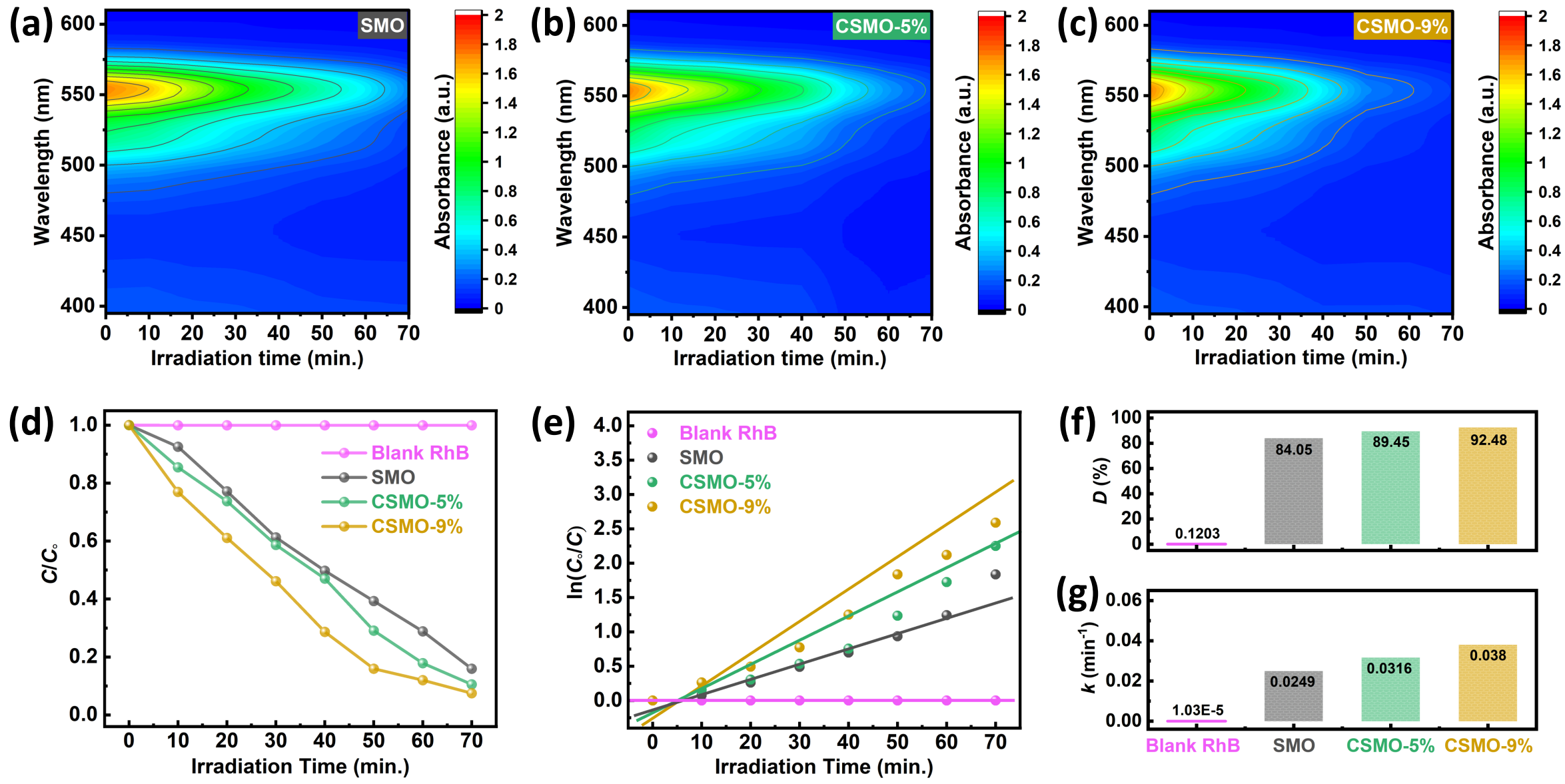

Figure S6. Contour plots of RhB dye degradation with (a) SMO, (b) CSMO-5%, (c) CSMO-9%, (d) correlation between C/C_o (e) rate coefficients with kinetic plot against irradiation time, (f) degrading efficiency, and (g) kinetic constants.

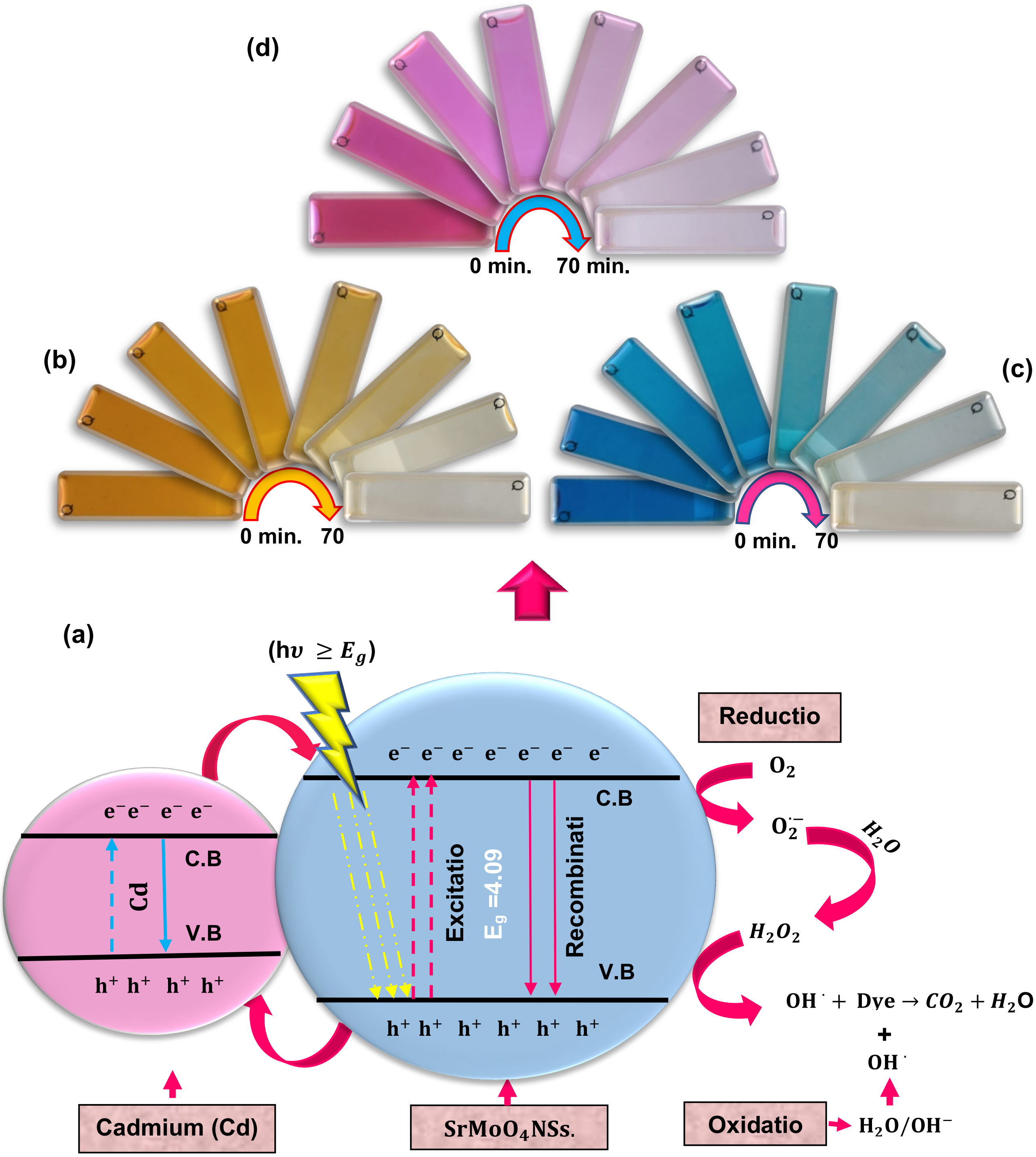

Figure S7. (a) Schematic illustration of the band structure of CSMO photocatalysts, the variation in colors of (b) MO dye, (c) MB dye and (d) Rh-B dye during 70 mins exposure of UV light in presence of CSMO-9% photocatalyst.

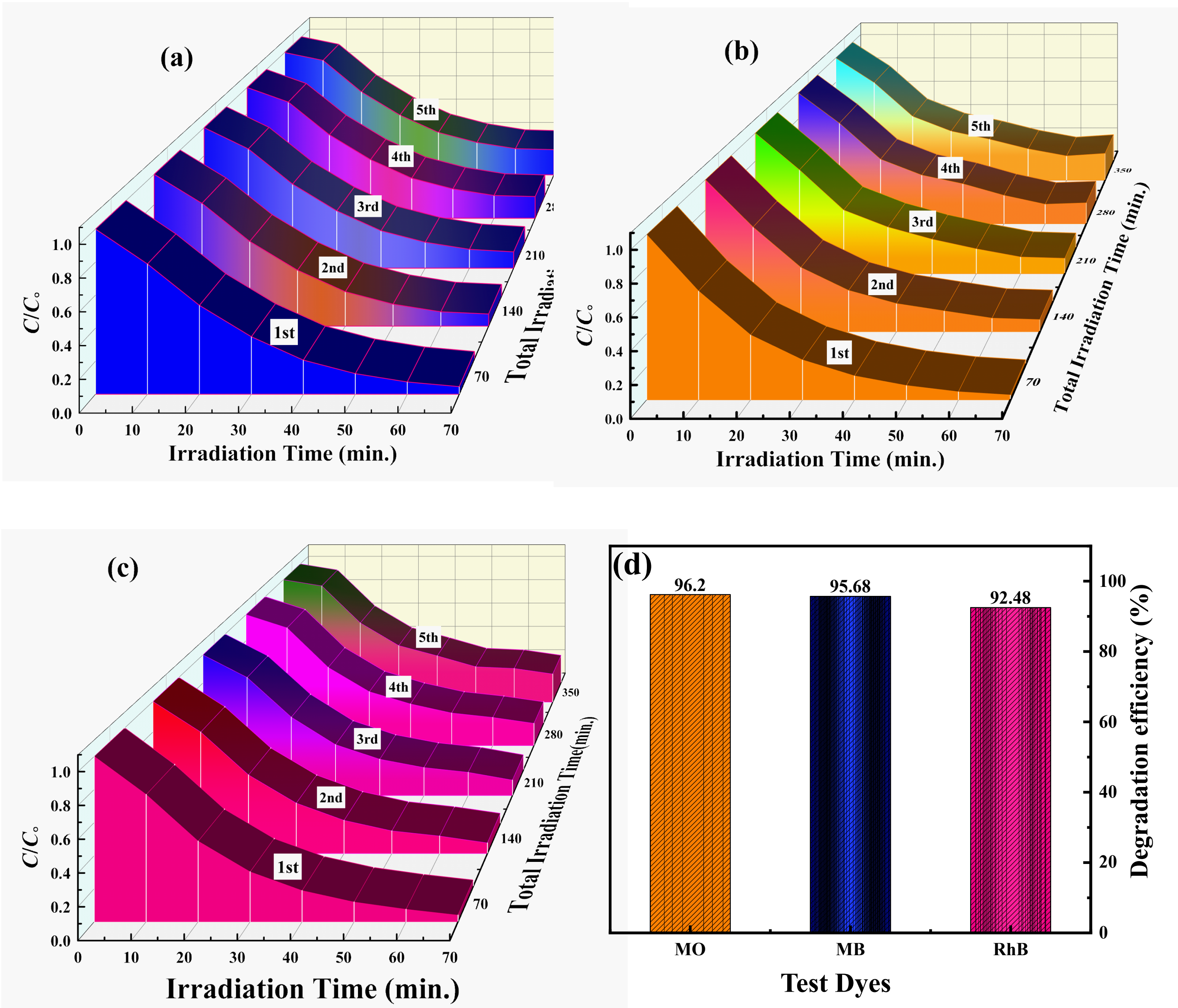

Figure S8. Durability assessment of the CSMO-9% in (a) MB, (b) MO, and (c) Rh-B degradation respectively and (d) Comparison of degradation efficiencies of CSMO-9% against MB, MO and Rh-B dyes.

References

S1. Seevakan, K.; Manikandan, A.; Devendran, P.; Shameem, A.; Alagesan, T., Microwave combustion synthesis, magneto-optical and electrochemical properties of NiMoO4 nanoparticles for supercapacitor application. *Ceram. Int.* **2018,** *44* (12), 13879-13887.

S2. Seevakan, K.; Manikandan, A.; Devendran, P.; Slimani, Y.; Baykal, A.; Alagesan, T., Structural, morphological and magneto-optical properties of CuMoO4 electrochemical nanocatalyst as supercapacitor electrode. *Ceram. Int.* **2018,** *44* (16), 20075-20083.

S3. Seevakan, K.; Manikandan, A.; Devendran, P.; Slimani, Y.; Baykal, A.; Alagesan, T., Structural, magnetic and electrochemical characterizations of Bi2Mo2O9 nanoparticle for supercapacitor application. *J. Magn. Magn. Mater.* **2019,** *486*, 165254.

S4. Seevakan, K.; Manikandan, A.; Devendran, P.; Baykal, A.; Alagesan, T., Electrochemical and magneto-optical properties of cobalt molybdate nano-catalyst as high-performance supercapacitor. *Ceram. Int.* **2018,** *44* (15), 17735-17742.

S5. Shameem, A.; Devendran, P.; Siva, V.; Packiaraj, R.; Nallamuthu, N.; Asath Bahadur, S., Electrochemical performance and optimization of α-NiMoO 4 by different facile synthetic approach for supercapacitor application. *J. Mater. Sci.: Mater. Electron.* **2019,** *30*, 3305-3315.

S6. Shameem, A.; Devendran, P.; Siva, V.; Murugan, A.; Sasikumar, S.; Nallamuthu, N.; Hussain, S.; Bahadur, S. A., Robust one-step synthesis of bismuth molybdate nanocomposites: A promising negative electrode for high end ultracapacitors. *Solid State Sci.* **2020,** *106*, 106303.

S7. Shameem, A.; Devendran, P.; Murugan, A.; Siva, V.; Bahadur, S. A., Synergistic effects of lanthanum nanoarchitecture in nickel molybdate nanospheres obtained by single-pot microwave synthesis and their applications in high-end asymmetric devices. *J. Phys. Chem. Solids* **2023,** *179*, 111392.

S8. Bhagwan, J.; Hussain, S. K.; Yu, J. S., Facile hydrothermal synthesis and electrochemical properties of CaMoO4 nanoparticles for aqueous asymmetric supercapacitors. *ACS Sustainable Chem. Eng.* **2019,** *7* (14), 12340-12350.

S9. Roshani, R.; Tadjarodi, A., Synthesis of ZnMoO4 nanofibers by ultrasonic bath method and investigation of their supercapacitive properties. *Mater. Today Commun.* **2021,** *26*, 101794.

S10. Veerasubramani, G. K.; Krishnamoorthy, K.; Sivaprakasam, R.; Kim, S. J., Sonochemical synthesis, characterization, and electrochemical properties of MnMoO4 nanorods for supercapacitor applications. *Mater. Chem. Phys.* **2014,** *147* (3), 836-842.

S11. Mai, L.-Q.; Yang, F.; Zhao, Y.-L.; Xu, X.; Xu, L.; Luo, Y.-Z., Hierarchical MnMoO4/CoMoO4 heterostructured nanowires with enhanced supercapacitor performance. *Nat. Commun.* **2011,** *2* (1), 381.

S12. Rani, B. J.; Swathi, S.; Yuvakkumar, R.; Ravi, G.; Rajalakshmi, R.; Al-Sehemi, A. G.; Velauthapillai, D., Samarium doped barium molybdate nanostructured candidate for supercapacitors. *J. Energy Storage* **2022,** *56*, 105945.

S13. Angelin, M. D.; Rajkumar, S.; Ravichandran, A.; Merlin, J. P., Systematic investigation on the electrochemical performance of Cd-doped ZnO as electrode material for energy storage devices. *J. Phys. Chem. Solids* **2022,** *161*, 110486.

S14. Deng, S.; Xiao, X.; Chen, G.; Wang, L.; Wang, Y., Cd doped porous Co3O4 nanosheets as electrode material for high performance supercapacitor application. *Electrochim. Acta* **2016,** *196*, 316-327.

S15. Farzi, M.; Moradi, M.; Hajati, S.; Toth, J.; Kazemzadeh, A., Synthesis of rod-like ternary Cu (Cd)-In-S and quaternary Cu-Cd-In-S by controlled ion exchange of MIL-68 (In) derived indium sulfide for high energy-storage capacitor. *Synth. Met.* **2021,** *278*, 116815.

S16. Ahmed, R.; Nabi, G., Morphology tailoring and enhanced electrochemical properties of Cd–Zn co-doped NiO nanorods for high performance supercapacitor. *Ceram. Int.* **2020,** *46* (14), 22330-22337.
S17. Sathiyan, A.; Rajkumar, S.; Dhineshkumar, S.; Merlin, J. P., Electrochemical performance of SrMoO4 as electrode material for energy storage systems. *J. Ind. Eng. Chem.* **2024,** *129*, 521-530.
S18. Zhu, Y.; Zheng, G.; Dai, Z.; Zhang, L.; Ma, Y., Photocatalytic and luminescent properties of SrMoO4 phosphors prepared via hydrothermal method with different stirring speeds. *Journal of Materials Science & Technology* **2017,** *33* (1), 23-29.
S19. Bazarganipour, M., Synthesis and characterization of BaMoO4 nanostructures prepared via a simple sonochemical method and their degradation ability of methylene blue. *Ceram. Int.* **2016,** *42* (11), 12617-12622.
S20. Gholami, A.; Maddahfar, M., Synthesis and characterization of barium molybdate nanostructures with the aid of amino acids and investigation of its photocatalytic degradation of methyl orange. *J. Mater. Sci.: Mater. Electron.* **2016,** *27*, 6773-6778.
S21. Hosseinpour-Mashkani, S. S.; Hosseinpour-Mashkani, S. S.; Sobhani-Nasab, A., Synthesis and characterization of rod-like CaMoO 4 nanostructure via free surfactant sonochemical route and its photocatalytic application. *J. Mater. Sci.: Mater. Electron.* **2016,** *27*, 4351-4355.
S22. Ray, S. K.; Kshetri, Y. K.; Dhakal, D.; Regmi, C.; Lee, S. W., Photocatalytic degradation of Rhodamine B and Ibuprofen with upconversion luminescence in Ag-BaMoO4: Er3+/Yb3+/K+ microcrystals. *J. Photochem. Photobiol., A* **2017,** *339*, 36-48.
S23. Gulzar, A.; Hassan, S.; Imran, Z., Exploring the photocatalytic activity of doped novel SrMoO4 nanoparticles in dye degradation: catalyzing change. *Ceram. Int.* **2024,** *50* (24), 54630-54642.
S24. Zhang, L.; Zheng, G.; Dai, Z.; Zhao, X., Synthesis of Co doping SrMoO4 for enhanced photocatalytic performance via hydrothermal method. *Dig. J. Nanomater. Biostruct* **2019,** *14*, 569-579.
S25. Huang, J.; Liu, H.; Zhong, J.; Yang, Q.; Chen, J.; Li, J.; Ma, D., Enhanced sunlight-driven photocatalytic performance of Bi-doped CdMoO4 benefited from efficient separation of photogenerated charge pairs. *Solid State Sci.* **2018,** *80*, 147-154.
S26. Phuruangrat, A.; Thongtem, S.; Thongtem, T., Effect of Ce dopant on photocatalytic properties of CaMoO4 nanoparticles prepared by microwave-assisted method. *Mater. Res. Innovations* **2022,** *26* (2), 84-90.
